# Design and Characterization of DNA-Driven Condensates: Regulating Topology, Mechanical Properties, and Immunorecognition

**DOI:** 10.1021/acsami.5c00428

**Published:** 2025-04-01

**Authors:** Elizabeth Skelly, Christina J. Bayard, Joel Jarusek, Benjamin Clark, Laura P. Rebolledo, Yasmine Radwan, Phong Nguyen, Melanie Andrade-Muñoz, Thomas A. Deaton, Alexander Lushnikov, Sharonda J. LeBlanc, Alexey V. Krasnoslobodtsev, Yaroslava G. Yingling, Kirill A. Afonin

**Affiliations:** † Chemistry and Nanoscale Science Program, Department of Chemistry, University of North Carolina at Charlotte, Charlotte, North Carolina 28223, United States; ‡ Department of Materials Science and Engineering, North Carolina State University, Raleigh, North Carolina 27695, United States; § Department of Physics, University of Nebraska Omaha, Omaha, Nebraska 68182, United States; ∥ Department of Physics, North Carolina State University, Raleigh, North Carolina 27695-8202, United States

**Keywords:** nucleic acid condensates, microrheology, dissipative particle dynamics, stimuli responsiveness, quantum dots, cGAS-STING

## Abstract

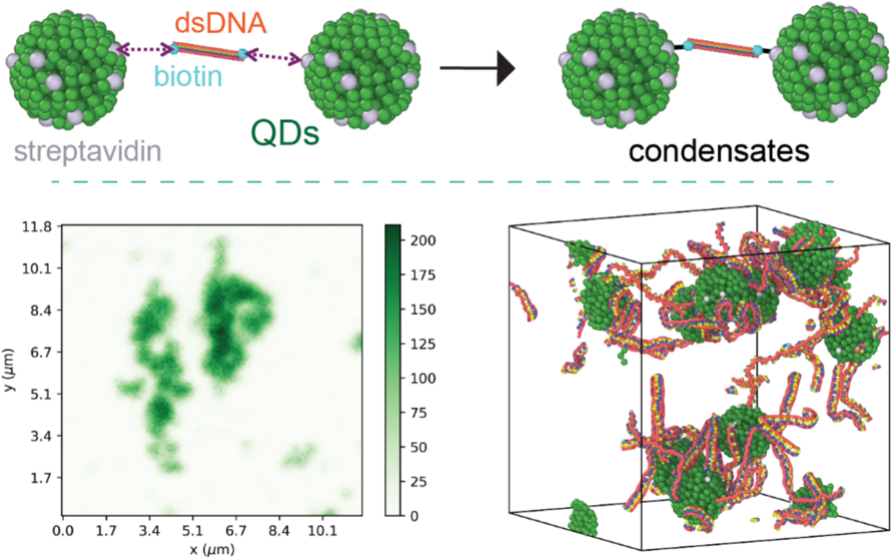

Cells maintain spatiotemporal control over biochemical processes through the formation and dissolution of biomolecular condensates, dynamic membraneless organelles formed via liquid–liquid phase separation. Composed primarily of proteins and nucleic acids, these condensates regulate key cellular functions, and their properties are influenced by the concentration and type of molecules involved. The structural versatility challenges the *de novo* design and assembly of condensates with predefined properties. Through feedback between computational and experimental approaches, we introduce a modular system for assembling condensates using nucleic acid nanotechnology. By utilizing programmable oligonucleotides and orthogonal synthesis methods, we control the structural parameters, responsive behavior, and immunorecognition of the products. Dissipative particle dynamics simulations predict some conditions to produce larger, well-defined condensates with compact, globular cores, while others result in smaller, more diffuse analogs. Fluorescence microscopy confirms these findings and microrheology demonstrates the viscoelastic adaptability of tested condensates. Nucleases trigger disruption of structures, and ethidium bromide intercalation protects condensates from digestion. Immunostimulatory assays suggest condensate-specific activation of the IRF pathway via cGAS-STING signaling. This study provides a framework for developing biomolecular condensates with customizable properties and immunorecognition for various biological applications.

## Introduction

With limited space but a multitude of biochemical processes, cells have achieved internal spatiotemporal control over biomolecules and their interactions. The formation and dissolution of biomolecular condensates allows for control over biochemical reaction kinetics.^1,2^ Biomolecular condensates are specific and recognizable micron-scale membraneless organelles within both prokaryotic and eukaryotic cells. These condensates have liquid-like features and serve as dynamic reaction centers for assemblies and synthesis of biomolecules.^3,4^ Condensates are unique systems formed through liquid–liquid phase separation (LLPS) with dense and dilute phases,^5^ and may include both homogeneous and heterogeneous multilayered structures that range from liquid- to partially solid-phases.^3^ Through LLPS, the formation of concentrated biomolecules allows for a wide range of biological effects to be enabled or enhanced, including transcriptional activation and repression, formation of granules and condensates, stress signaling, and viral assembly.^6−9^

Physical properties of condensates, such as viscoelastic characteristics and stimuli responsiveness, are strongly affected by the type of biomolecules that enter their composition.^3^ Many condensates contain nucleic acids, which promote phase separation; however, high ratios of RNA to protein within the condensates can inhibit phase separation. Thus, controlling the concentration of biomolecules in the cellular environment allows for controlled phase transitions.^5,10−13^ Regulation can also occur through biomolecule recruitment timing in response to stimuli or during different cell cycle stages.^14−16^ Assembly of biomolecular condensates can be initiated by oversaturation and overexpression of biomolecules.^3^ The capacity of biomolecular condensates to generate numerous weak interactions, such as hydrophobic attraction, hydrogen bonding, and electrostatic interactions, becomes a key aspect of condensates’ ability for phase separation.^4,17^

Nucleic acid nanotechnology offers a powerful approach for engineering programmable condensates with precise control over their properties, where canonical Watson–Crick base pairing dictates the predictable organization of various materials.^2,18−20^ Our previous studies demonstrated the precise control over the nucleic acid-driven 3D organization of quantum dots (QDs), used as a model system.^18^ However, several key challenges remain, such as developing methods to introduce controlled flexibility and responsiveness into nucleic acid–based condensates, controlling condensate properties across multiple length scales, understanding the relationship between structural parameters and formation kinetics, stability, and phase behavior. Additional challenges include fine-tuning mechanical properties, creating condensates capable of dynamic responses to multiple stimuli, incorporating diverse functional elements without compromising activity and specificity, and translating the precise control achieved in vitro to cellular and in vivo environments.^21,22^ Addressing these challenges is essential for designing programmable assemblies to mimic and surpass the capabilities of natural condensates.

Understanding the complex interactions within biomolecular condensates across different scales has been greatly advanced by computational methods, including molecular simulations and machine learning (ML).^23−30^ All-atom molecular dynamics (AMD) simulations have provided high-resolution insights into specific interactions and dynamics within condensates, revealing the structure of dense and dilute phases in systems such as elastin-like peptides and intrinsically disordered proteins.^23,31^ Chen et al.^32^ utilized AMD for the development of DNA origami methods for the spatial control of QDs, enabling the creation of precise and programmable 3D nanostructures. While AMD provides atomic-level detail and insight into specific interactions, its high computational costs restrict simulations to small systems and short time scales, making it challenging to study large, dynamic systems of biomolecular condensates. To address these limitations, coarse-grained (CG) models have been used to reduce computational demands and explore broader temporal and spatial scales,^33^ with tools like OpenABC^34^ streamlining the setup of CG simulations of condensates. Dignon et al. demonstrated that CG molecular dynamics can effectively model phase behavior accounting for variables such as temperature and salt concentration.^35^ Another CG technique, Dissipative Particle Dynamics (DPD),^36^ has been utilized to study mesoscale behavior in systems such as colloidal suspensions, polymer melts, phase-separating fluids, and inorganic materials.^36^ Recently, Chandler et al.^18^ employed DPD to explore how nucleic acids act as cross-linkers for QD assembly into organized 3D nanostructures.

ML methods have recently emerged as a powerful tool for predicting protein phase behavior. Sequence-based models like FuzDrop^37^ and PSPredictor,^38^ as well as structure-based predictors such as DeePhase^39^ and PhaSepDB,^40^ enable rapid proteome-wide screening for phase-separating proteins. CGnet, a class of neural networks developed to predict the CG mean force and free energy as well as coarse-graining all-atom models, represents an emerging area of elegant ML solutions to simulation problems still in its infancy and not yet transferable to other systems.^41^ However, despite these advancements, ML models remain predominantly predictive and lack the real-time simulation capabilities required to capture dynamic phase behaviors and interactions within condensates.^42^

**Figure 1:**
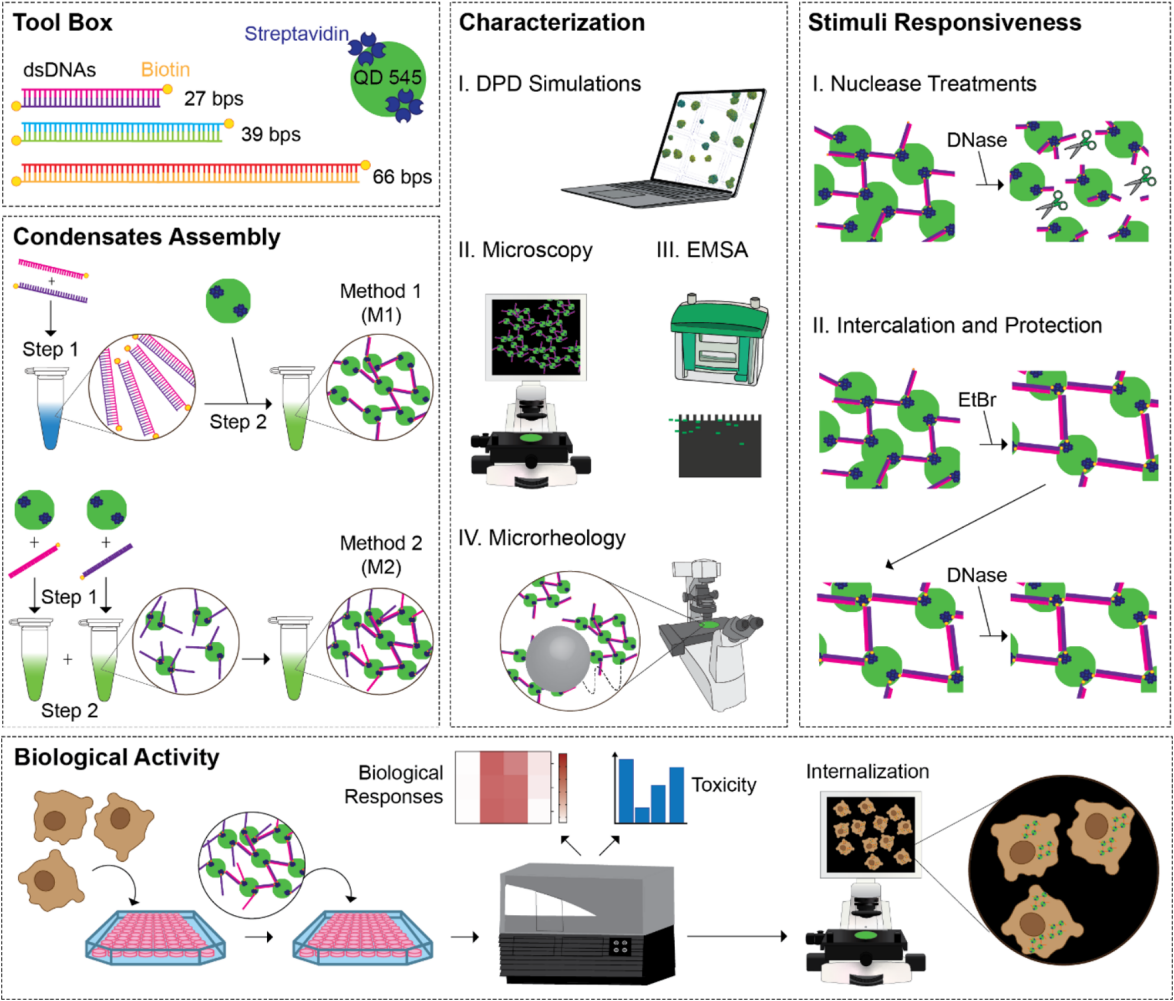
Experimental pipeline of assembly and characterization of the biomolecular condensates described in this work.

In this work, schematically depicted in Figure 1, we introduce a user-friendly experimental pipeline that relates the architectural variables, production protocols, and the presence of stimuli with the ability to form condensates exhibiting regulated properties and responsive behavior, as predicted *in silico*. The customizable design of short oligonucleotides allows reconfiguration through interactions with external mechanical and biochemical stimuli, such as the presence of the nucleases, changes in salt concentration, mechanical stress, and intercalation. Furthermore, we demonstrate that the rational design of programmable condensates allows us to regulate their immunorecognition by human cells. The current study combines continuous feedback between different computational components and experimental data to synergize the overall refinement of the condensates’ design and production protocols. The developed principles can be readily applied to expand the design capabilities of other new classes of responsive biomaterials for a broad range of applications.

## Materials and Methods

### Computational Studies

The DPD simulations were performed using LAMMPS software.^43−45^ All details and additional information on DPD can be found in Supporting Information. An overview of the DPD force field and LAMMPS parameters is given in Table S1. A single trajectory was collected for each condition. This DPD system uses a two-bead approach for nucleic acids where the “backbone” bead comprises of the sugar ring and phosphate group with inherent negative charge, while the “base” bead consists of unspecified nucleobases. An angular potential was included in building each single-stranded DNA (ssDNA) strand to create the preference for a helical shape upon hybridization with complementary ssDNA. Therefore, two angular potentials were mapped from the atomistic MD simulations capturing the angles of (1) the center of mass along the backbone (θ_0_ = 150°) and (2) between the backbone and nucleobase (θ_0_ = 100°). The calculation of the angular potential was harmonic
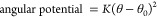
with a harmonic constant, *K*.

**Figure 2:**
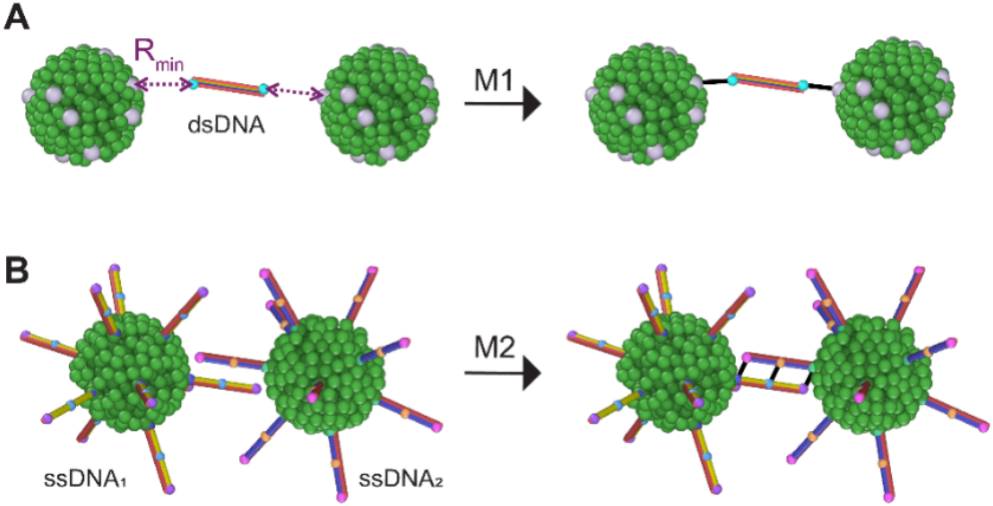
Overview of dyBonding implementation. (A) In M1, the QDs (green) have ″streptavidin″ beads (gray) on the surface and there are ″biotin″ beads (cyan) on the ends of dsDNA strands, allowing streptavidin–biotin interactions when they are within the critical distance, *R*_min_. (B) In M2, each ssDNA strand has three reactive beads on antiparallel strands to allow for the complementary strands present on separate QDs to fully hybridize, bringing QDs together.

For condensate assembly via method 1 (M1), the system comprises 10 QDs and 50 double-stranded DNAs (dsDNAs) randomly placed in the simulation box. Alternatively, method 2 (M2) consisted of 10 QDs, half of which were decorated with 10 ssDNAs of base type 1 (ssDNA_1_) while the other half were decorated with 10 complementary ssDNAs of base type 2 (ssDNA_2_), as shown in Figure 2. For both methods, the length of the DNA strands was modeled for 27, 39, and 66 base pairs (bps), which were used in all experimental studies. The QDs were composed of 336 beads, randomly placed with PACKMOL into a diameter of 6*r*_C_, and systems were created by randomly placing QDs and DNA in a simulation box with in-house MATLAB scripts. Each QD was fixed in LAMMPS as a rigid body so that forces and torques were calculated as the sum of each rigid body’s constituent particles to allow the body to move and rotate as a single entity. The periodic system volume varied from 30*r*_C_^3^ for the 27 bps systems to 38*r*_C_^3^ for the 66 bps system to fix the concentration around 10%. A DPD bead density of 3 was used and each simulation was executed for 8 × 10^5^ timesteps. The convergence of simulations was assessed by monitoring the reduction in the number of bonds formed during each simulation, indicated by a change in slope, as illustrated in Figure S3.

For each DNA length, the solvent ionic strength was varied through the implicit solvent ionic strength DPD method.^46^Table S2 presents the interaction parameters (*aij*) for various pairs of components, including DNA backbone, nucleobases, QDs, and water. The interaction strengths were carefully tuned to reflect the physical properties of the system and were based on previous computational work on almost identical QD-DNA systems, which varied the backbone–backbone interactions to explore the effects of salt concentration.^18^ For instance, the backbone–backbone interactions were set to a high value (*aij* = 90) to represent the strong electrostatic repulsion between DNA strands, which was reduced (*aij* = 40) under high salt conditions to account for charge screening effects. The interactions between complementary nucleobases (type A to type B) were set to a low value (*aij* = 5) to promote base pairing, while self-interactions of nucleobases (type A to type A) were stronger (*aij* = 22) to prevent nonspecific aggregation. Importantly, the interactions between DNA components and QDs were parametrized to capture the experimentally observed behavior of DNA-QD conjugates. The backbone-QD interaction was set to a relatively low value (*aij* = 15) to allow for DNA wrapping around QDs, while the nucleobase-QD interaction was stronger (*aij* = 27) to represent potential π-π stacking interactions. This parametrization scheme allowed our model to capture the essential physics of DNA-QD interactions within condensates while maintaining computational efficiency. By employing such a coarse-grained approach, we were able to simulate larger systems over longer time scales, enabling us to study the formation and properties of biomolecular condensates at biologically relevant scales.

Our DPD dynamic bonding (dyBonding) method allows for the real-time formation of permanent bonds during simulations based on user-defined nucleotide type, distance, and time criteria. Here, dyBonding was employed to model two distinct mechanisms of condensate formation: QD cross-linking using double-biotinylated dsDNAs (M1) and hybridization of ssDNAs linked to individual QDs (M2). For M1, the biotin/streptavidin interaction was modeled by representing biotin as a single bead attached to the end of dsDNA, while streptavidin was represented by 10 beads placed on the QD surface. The nonbonded interactions for biotin beads were set identically to backbone beads, with a low repulsive parameter of 5 between biotin and streptavidin beads to allow for bond formation. In M2, the hybridization of ssDNA was simulated by placing three reactive beads on each ssDNA strand, arranged in an antiparallel configuration to their complementary strands. Similar to M1, a low repulsive parameter of 5 was assigned between beads that were allowed to form bonds.

This dyBonding approach, combined with the carefully parametrized nonbonded interactions, provides a powerful framework for investigating the formation, structure, and properties of biomolecular condensates. By allowing for the dynamic formation of bonds during simulation, dyBonding captures the essential process of condensate assembly through both protein–ligand interactions and nucleic acid hybridization. This method bridges the gap between molecular-scale interactions and mesoscale condensate properties while maintaining the ability to simulate specific molecular recognition events crucial for condensate assembly.

Clustering analysis of each simulation was performed using Density-Based Spatial Clustering of Applications with Noise (DBSCAN) within the Python Scikit-learn package.^47^ The maximum distance for beads to be considered in the same neighborhood (eps) was set to 6 (the diameter of the QDs in LAMMPS) and the minimum number of samples in a neighborhood was set to 169 (half of the number of DPD beads per QD + 1).

### Preparation of Condensates

The term ″condensates″ in our work refers to the mimicking of nonmembrane-bound compartments that concentrate specific biomolecules, such as proteins and nucleic acids. QDs facilitate protein–DNA interactions through the streptavidin molecules on their surface, which bind to biotinylated DNA. Additionally, the inherent fluorescence of QDs enables tracking the formation of condensates. Biotinylated ssDNAs were purchased from Integrated DNA Technologies (IDT).^18,48^ All sequences are listed in the Supporting Information. ssDNAs were resuspended in double deionized water (ddiH_2_O) before use. The streptavidin-decorated QDs were purchased from ThermoFisher (Qdot 545 ITK Conjugate Kit).

For M1 (QDs + dsDNAs) - following previous protocols,^18^ the double-biotinylated dsDNAs were formed through the combination of two complementary ssDNA_1_ and ssDNA_2_ at a 1:1 molar ratio. The samples were vortexed, centrifuged and incubated at 95 °C for 2 min, followed by the addition of the assembly buffer (a final concentration of 89 mM tris-borate (pH 8.2), 50 mM KCl, and 2 mM MgCl_2_). The samples were then incubated at 25 °C for 20 min. A molar ratio of 1:5 QDs:dsDNAs was combined, vortexed, and centrifuged before incubation at 37 °C for 30 min. The samples were stored at 4 °C.

For M2 (QDs-ssDNA_1_ + QDs-ssDNA_2_) - following previous protocols,^18^ streptavidin-decorated QDs were mixes with biotinylated ssDNAs in 1:10 molar ratio, separately and incubated at 37 °C for 20 min. In a separate tube, a 1:1 (QDs-ssDNA_1_):(QDs-ssDNA_2_) molar ratio was combined, vortexed, centrifuged and incubated at 37 °C for 30 min. The samples were stored at 4 °C.

All condensates were analyzed using electrophoretic mobility shift assays (EMSA) on 2% agarose gels with ethidium bromide (EtBr). The gels were run for 20 min at 220 V and then visualized with Bio-Rad ChemiDoc MP Imaging System.

### Kinetics Assays for Condensates Formation

Right after the mixing, the condensate samples (QDs + dsDNAs, for M1 and QDs-ssDNA_1_ + QDs-ssDNA_2_, for M2) were incubated at 37 °C and 4 μL were aliquoted and snap frozen on dry ice at each time point: 0.5, 1, 5, 15, and 30 min. After 30 min, 1 μL of DNase (Sigma-Aldrich) was added and the mix was additionally incubated for 60 min at 37 °C. All samples were loaded on 2% agarose gel with EtBr in reverse order, along with free QDs and duplexes as controls. The gel was visualized as described above.

### Microrheology

The optical setup for passive microrheology studies was constructed around IX-73 Olympus inverted microscope, equipped with a 100× objective lens (air) and a Sentech CCD 4.0 MP variable-scan-speed camera. The setup was calibrated using a set of glycerol/water mixtures with known viscosity values, η, following previously established protocols.^49^ To evaluate the viscoelastic characteristics of synthetic biomolecular condensates, the movement of the microbeads was examined over a duration of two to three seconds. 0.5 μL of the stock 2 μm polystyrene bead solution (Sigma-Aldrich, Co.; actual average diameter of the beads *d* = 1.92 μm) were mixed with 50 μL of preassembled condensates at 2 μM DNA, 0.2 μM QDs, and immediately placed (∼30 μL) in microchamber for observation. After focusing the objective lens on the beads, 40 s videos of the motion of various beads were recorded with 25 frames per second video rate. Multiple beads were captured in each video to ensure robust statistical analysis. The bead motion was then tracked using Fiji (ImageJ) software package and the Mosaic plugin using the Single Particle Tracking Analysis option.^49^ The tracking of a bead’s motion yields a trajectory showing the distance traveled by the bead in two dimensions, Δ*r (*Δ*x*, Δ*y)*, in nanometers versus time, *t*, in seconds. To extract rheological information about the condensates, the ensemble-averaged mean-squared displacement (MSD), < Δ*r*^2^>, was calculated as a function of lag time, τ, using the following equations: Δ*r*^2^(*i*) = Δ*x*^2^(*i*) + Δ*y*^2^(*i*) and < Δ*r*^2^(τ)> = <Σ(*r*_*i*+*n*_ – *r*_*i*_)^2^>, where *n* = 1, 2, 3··· *N*-10 and *N* is the total number of frames in the video. The MSD plots were then fitted using
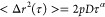
and setting *p* = 2 (for the 2-dimensional case) with *D* and α as fitting parameters.^49^ The power of the lag time, α, was treated as an identifier of the viscoelastic state of the material.

### Time-Resolved Fluorescence Microscopy

The microscope used for time-resolved fluorescence microscopy was a MicroTime 200 (PicoQuant, Berlin) with SymPhoTime64 software for data acquisition. The sample was excited with a 531 nm picosecond pulsed diode laser, driven by a laser driver module (SEPIA II). The laser was focused onto the sample with a 60× 1.2 numerical aperture (NA) water immersion lens (Olympus UPlanSApo, Superachromat). A fast galvo beam scanning module (FLIMbee) was used to control laser beam scanning. Fluorescence from the sample was collected with the same objective, and spatially filtered through a circular 100 μm confocal pinhole. A single photon avalanche diode (SPAD – Excelitas) was used to detect the sample fluorescence with a 582/64 bandpass filter. A multichannel event timer (MultiHarp 150) time tagged individual photons detected at the SPAD, relative to the laser pulse (nanotime) and relative to the start of the experiment (macrotime) using time correlated single photon counting (TCSPC). Fluorescence lifetime imaging microscopy (FLIM) images were produced by plotting the average photon arrival nanotime at each image pixel. Fluorescence correlation spectroscopy (FCS) was performed by binning macrotimes in 1 ms bins and autocorrelating the resulting fluorescence intensity signal. The resulting autocorrelation curves were fit to an appropriate model to extract a diffusion coefficient.

For time-resolved fluorescence imaging of condensates, multichannel flow cells were built by sandwiching a quartz slide and glass coverslip that were chemically functionalized with a solution of 1% biotin-methoxy-PEG (Laysan Bio Biotin-PEG-SIL-3400, mean *M*_w_ of 3.4 kDa), and 99% methoxy-PEG (Laysan Bio MPEG-SIL-2000, mean *M*_w_ of 2 kDa). The channels were rinsed with a binding buffer (20 mM Tris HCl, 5 mM MgCl_2_, 100 mM sodium acetate, pH 7.5). A 10 nM solution of the condensate sample diluted in image buffer [binding buffer containing an enzymatic oxygen scavenging system (2% glucose and 1000 U/ml catalase) and triplet state quencher (0.02% cyclooctatetraene)] was injected and incubated for several minutes to allow the remaining streptavidins in condensates to attach to the biotinylated coverslip. The channel was flushed again with an image buffer to remove unbound condensates. The laser power was 3.5 μW measured before the objective lens.

Following fluorescence imaging of condensates prepared by both assembly methods, ImageJ was used to evaluate the aspect ratios of each condensate. The aspect ratio is the width divided by the length of the sample, determined by ImageJ. The longest side of the condensate (length) was measured first, then the shorter side (width) perpendicular to the length was measured. The closer the aspect ratio was to 1, the rounder the morphology. Both measurements were graphed using GraphPad Prism.

### Atomic Force Microscopy (AFM)

Freshly cleaved mica was modified with 1-(3-aminopropyl)-silatrane (APS) as previously described.^50−52^ Briefly, small pieces of mica were cleaved using Scotch tape, immersed in an APS solution (C_APS_ = 167 μM) for 30 min, and dried with ultrahigh-purity argon. The samples were diluted in assembly buffer (89 mM tris-borate (pH 8.2), 50 mM KCl, and 2 mM MgCl_2_) and deposited onto APS-modified mica. After a 2 min incubation, the samples were briefly rinsed with several drops of deionized water and dried using a gentle flow of argon. Images were collected with a MultiMode 8 system (Bruker Instruments) in PeakForce Tapping Mode under ambient conditions. Silicon probes (MSNL-E, Bruker Nano Inc.) were used for imaging at a scanning rate of approximately 1 Hz. Image processing was performed using the FemtoScan software package (Advanced Technologies Center, Moscow, Russia).

### Stimuli Responsiveness Studies

Once condensates were formed using the above methods, different stimuli were added. For DNase digestion, condensates were diluted to 9 nM in binding buffer with 0.09 mg/mL DNase. The solution was incubated for 15 min. The DNase digested condensates were then imaged using the time-resolved fluorescence imaging method measurement method described. For EtBr intercalation, condensates were diluted to 10 nM in binding buffer with 6.18 μg/mL EtBr. The condensates were incubated for 30 min at room temperature. The intercalated EtBr condensates were then imaged using the time-resolved fluorescence imaging method described. For EtBr intercalation prior to DNase digestion, condensates were diluted to 10 nM in binding buffer with 6.18 μg/mL EtBr. The condensates were incubated for 30 min at room temperature. To the intercalated EtBr condensate sample, DNase was introduced and incubated for 30 min at 37 °C. Post EtBr intercalation and DNase digestion, the EtBr condensates were imaged using the time-resolved fluorescence imaging method described.

### Immunostimulatory Responsiveness

NF-κB–SEAP and IRF–Lucia Reporter cells (THP1-Dual reporter cells from InvivoGen) were thawed following the manufacturer’s protocol in media composed of RPMI containing 20% heat-inactivated fetal bovine serum and were maintained at standard conditions of 37 °C and 5% CO_2_. Cells were passaged twice prior to experiments. Cells were sustained using two media types: maintenance and growth media. Maintenance media was RPMI 1640, 2 mM l-glutamine, 25 mM HEPES, 10% heat-inactivated fetal bovine serum (30 min at 56 °C), 100 μg/mL Normocin, Pen-Strep (100 U/mL–100 μg/mL). Growth media was maintenance media with the addition of 10 μg/mL Blasticidin and 100 μg/mL Zeocin. Both media were used interchangeably in every other passage. Before experiments, the cells were counted using a hemocytometer and Countess Cell Counter and seeded at a density of 100 K cells/well in a 96 well Greiner plate, with a final volume of 100 μL/well using test media (RPMI 1640, 2 mM l-glutamine, 25 mM HEPES, 10% heat-inactivated fetal bovine serum, Pen-Strep (100 U/mL–100 μg/mL) without Blasticidin, Normocin and Zeocin). Freshly prepared condensates were complexed with Lipofectamine 2000 (L2K) and incubated at 25 °C for 20 min. The cells were then transfected with 1, 5, or 10 nM (by final DNA) condensates. Cell viability, as well as SEAP and Lucia secreted activities were assessed after 24 h using MTS, Quanti-Blue, and Quanti-Luc assays, respectively, following the InvivoGen protocols.

Also, NF-κB-SEAP reporter HEK293 cells expressing human toll-like receptor 9 (hTLR9) cells (HEK-Blue hTLR9 cells from InvivoGen) were thawed following the InvivoGen protocol in DMEM, 4.5 g/L glucose, 2 mM l-glutamine, 10% (v/v) fetal bovine serum, 100 U/mL penicillin, 100 μg/mL streptomycin, 100 μg/mL Normocin and passaged twice prior to experiments. Cells were plated at 100 K cells/well. Condensates complexed with L2K were transfected at a final concentration of 10 nM (by DNA). Cell viability was monitored using MTS assay and SEAP activity was evaluated using Quanti-Blue assay using a Tecan Spark microplate reader.

### Evaluation of cGAS Binding to dsDNAs

Plasmid containing cGAS was obtained from Addgene and cGAS expression and purification were followed as described elsewhere with modification.^53^ Briefly, His_6_-tag and SUMO tag were not removed before dialysis and no further fractionation or size exclusion chromatography were performed. cGAS collected after dialysis was adjusted to a final concentration of 3 mg/mL in 50% (v/v) glycerol. dsDNAs of 27, 39, and 66 bps were tested for relative cGAS binding. Four pmol of DNA and 15 μg cGAS were combined and incubated at 37 °C for 10 min. The same amount of DNA with and without cGAS was loaded on 3.5% agarose TBE gel. Electrophoresis was carried out in cold buffer at 300 V and room temperature for 10 min and then the gels were stained with EtBr for 15 min and imaged.

### Cellular Uptake of Condensates

MDA-MB-231 cells were cultured in DMEM supplemented with 10% heat-inactivated fetal bovine serum (FBS) and 1% penicillin-streptomycin (100 U/mL–100 μg/mL) and maintained at 37 °C with 5% CO_2_. For uptake experiments, cells were seeded in a 24-well flat-bottomed Greiner plate at a density of ∼ 80,000 cells per well in a 400 μL plating volume. After 24 h to allow for adherence, the cells were transfected with condensates, either complexed with or without L2K. Treatments (100 μL) were prepared to reach a final volume of 500 μL per well. At 24 h post-transfection, cells were imaged using a Leica DMi8 inverted fluorescence microscope before flow cytometry analysis. For flow cytometry, supernatant (500 μL) was collected into an Eppendorf tube, and cells were detached with 250 μL of 0.25% trypsin-EDTA at 37 °C, 5% CO_2_ for 5 min. Trypsinization was stopped by adding 250 μL of DMEM supplemented with 10% FBS and 1% penicillin-streptomycin. The cell suspension (500 μL) was then combined with the collected supernatant and centrifuged at 600 rcf for 5 min. The supernatant was aspirated and replaced with 600 μL of 1× PBS containing 4% bovine serum albumin (BSA) and 0.2 mM EDTA before analysis on an Attune NxT Flow Cytometer. Uptake efficiency was determined using the OVERTON analysis.

## Results and Discussion

To evaluate the properties of the biomolecular condensates and their responses to various stimuli, we employed complementary techniques, including computational simulations, EMSA, fluorescence microscopy, and microrheology.

**Figure 3:**
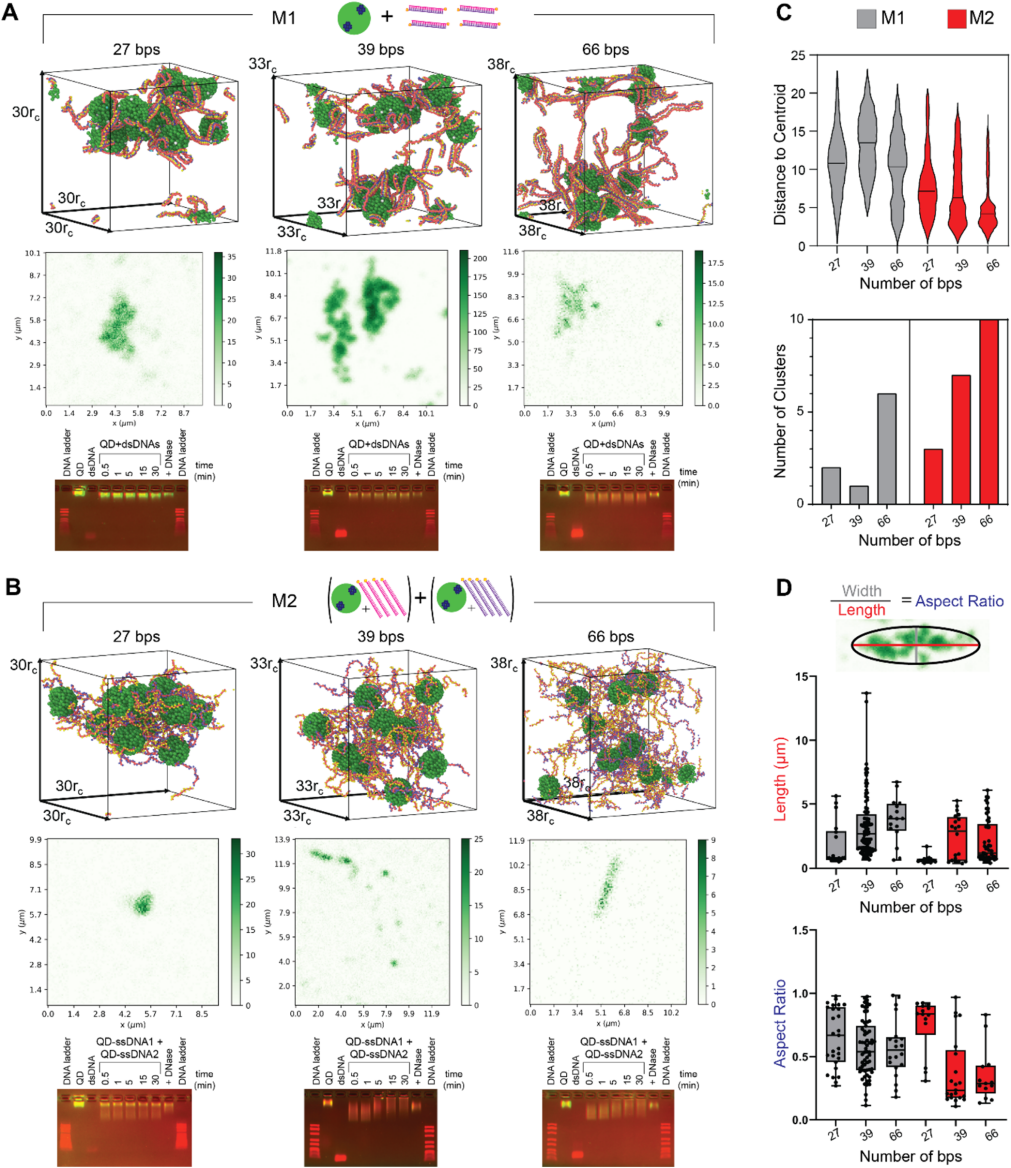
Characterization of condensates through computational modeling, fluorescence microscopy, and electrophoretic mobility shift assays. DPD models and corresponding representative fluorescent microscopy images and EMSAs of (A) M1 and (B) M2 condensates with different DNA lengths. (C) Using DPD simulations, the distance from the centroid was calculated and plotted, showing the distribution of sizes for each sample type. (D) The aspect ratio found for each condensate from microscopy by measuring the length of the longest side of the condensate and the width of the condensate perpendicular to the length. Figure S5 shows further fluorescence intensity images and representative experimental decays.

Our computational studies, (Figure 3) suggest that M1 rapidly produces larger condensates with more compact cores that appear well-defined and more globular. In contrast, M2 results in a slower formation of a higher number of smaller, more linear, fragmented and dispersed condensates, which are distributed more evenly throughout the system. This was confirmed experimentally and the difference in condensate size and distribution became evident across all DNA lengths.

To determine condensate “spread”, the distances from each point to its cluster center were recorded and compared. The data distribution was determined to be non-normal based on the Asymptotic one-sample Kolmogorov–Smirnov test at a significance level of 5% and significant, systematic deviations in the Q-Q plots for each sample. Despite non-normality, the Welch two-sample *t* test was employed due to its robustness with large data sets, alongside the Wilcoxon rank sum test, which is commonly used for non-normal data. As shown in Table 1, both tests yielded p-values of less than 2.3 × 10^–16^, indicating substantial statistical differences between methods 1 and 2 for all lengths examined. A higher test statistic (t) indicates a larger difference in the means, with the 39 bps condensates having the greatest differences between methods, followed closely by the 66 bps and then the 27 bps condensates. The 95% confidence interval for the 27 bps comparisons reveals that, on average, method 1 produces condensates that are 2.84 to 3.09*r*_C_ larger. The 39 bps condensates had the largest difference, with the 95% confidence interval for the differences between methods 1 and 2 revealing that method 1 produced condensates that were an average of 5.80 to 6.05*r*_C_ larger.

**Table 1:** Statistical Analysis of DBSCAN Clustering Performed on Final Frame of DPD Simulations

			Welch two-sample *t* test			Wilcoxon rank sum test
sample M# (# of bps)	# of clusters	avg. distance to cluster centroid (avg. across clusters)	test statistics (t)	95% confidence interval	*P*-value	*P*-value
M1 (27)	2	10.81	46.18	(2.84, 3.09)	2.2 × 10^–16^	2.2 × 10^–16^
M2 (27)	3	7.84				
M1 (39)	1	13.29	94.15	(5.80, 6.05)	2.2 × 10^–16^	2.2 × 10^–16^
M2 (39)	7	7.36				
M1 (66)	6	9.89	81.40	(4.51, 4.74)	2.2 × 10^–16^	2.2 × 10^–16^
M2 (66)	10	5.27				

Figure S1 presents the final frames for all DNA length and method combinations simulated at high salt concentration, as well as an overall comparison of low and high salt conformations for both methods. Figure S2 shows the distribution of cluster sizes and the final number of clusters for all high salt concentration simulations. The DBSCAN clustering analysis (Table S3) revealed no consistent trend across lengths for M1. While the condensates formed with 27 and 66 bps presented significantly more compact structures compared to M2, the 39 bps condensates formed via M1 were more spread out than their counterparts from M2. M2 consistently formed a single cluster across all lengths, while M1 produced more clusters with a denser core at high salt concentration compared to low for all lengths except 66 bps.

At high salt concentration, both methods yielded more compact structures (also observed experimentally, Figure S4), with M1 favoring smaller, more numerous globular clusters and M2 creating a more porous network. This structural tightening can be attributed to the reduced bead self-repulsion (*a*_*ii*_) at higher salt concentration, promoting greater ssDNA hybridization in M2 and enhanced biotin–streptavidin bead interactions in M1. However, these results warrant further experimental verification, as the behavior observed at high salt could be an artifact of the dyBonding method and DPD simulations.

To verify the complete assembly of the condensates, EMSAs were conducted, separating the samples based on size and charge. Free streptavidin-decorated QDs did not initially move through the gel, whereas negatively charged DNAs rapidly migrated toward the positive electrode. The increase of overall negative charge of individual QDs upon ssDNA binding made the complexes move toward the positive electrode. However, when condensates formed, the large size limited them from entering the gel, resulting in a stuck-in-the-well appearance. Different ratios of QD to DNA concentrations were used to determine the optimal conditions for condensate formation without excess DNA. The optimal QD:DNA ratio was found to be 1:5 for M1 and 1:10 for M2. Since M1 relies on double-biotinylated dsDNAs, their binding streptavidin-coated QDs drives the rapid formation of condensates. In contrast, M2 involves separately mixing cognate QDs decorated with complementary ssDNAs, resulting in a slower assembly process. Kinetic studies indicated that condensate formation was complete within the first minute for M1, whereas M2 required 15 min of incubation (Figure 3A,B)

Confocal microscopy revealed that the resulting condensates post assembly were heterogeneous in size and morphology (Figures 3 and S5). The differences coincided with the lengths of DNAs, method of assembly and the conditions during formation. Based on aspect ratio measurements, the roundest (aspect ratio ∼1) condensates were mostly observed in M2 for DNA lengths of 27 bps, followed by M1 condensates of the same DNA lengths. M2 condensates composed of 39 and 66 bps demonstrated the least round (aspect ratio <1) morphologies.

To assess the local mechanical properties of condensates, we utilized particle tracking (PT) microrheology. This experimental method analyzes thermally induced motion of microbeads embedded in a sample to determine its viscoelastic characteristics.^54^ PT microrheology is advantageous due to its minimal sample volume requirements and straightforward preparation, allowing rapid and reliable results. The observed responses, linear or nonlinear, depend on the system’s viscoelastic properties.

The multicomponent nature of biomolecular condensates leads to their ability to form extensive cross-linking with a substantial contribution of the liquid phase still diffusing through the system. As such, the condensates are expected to exhibit a complex interplay of viscous and elastic properties. In passive PT microrheology, the motion of microscopic beads is tracked over time to assess the sample’s spatiotemporal rheological properties. The motion reflects how the fluid environment affects particle diffusion, offering insights into the material’s viscosity and elasticity. In this study, 2 μm beads were embedded in biomolecular condensates where their thermal fluctuations were small enough not to cause any structural rearrangement of the condensate structure.^55^

**Figure 4:**
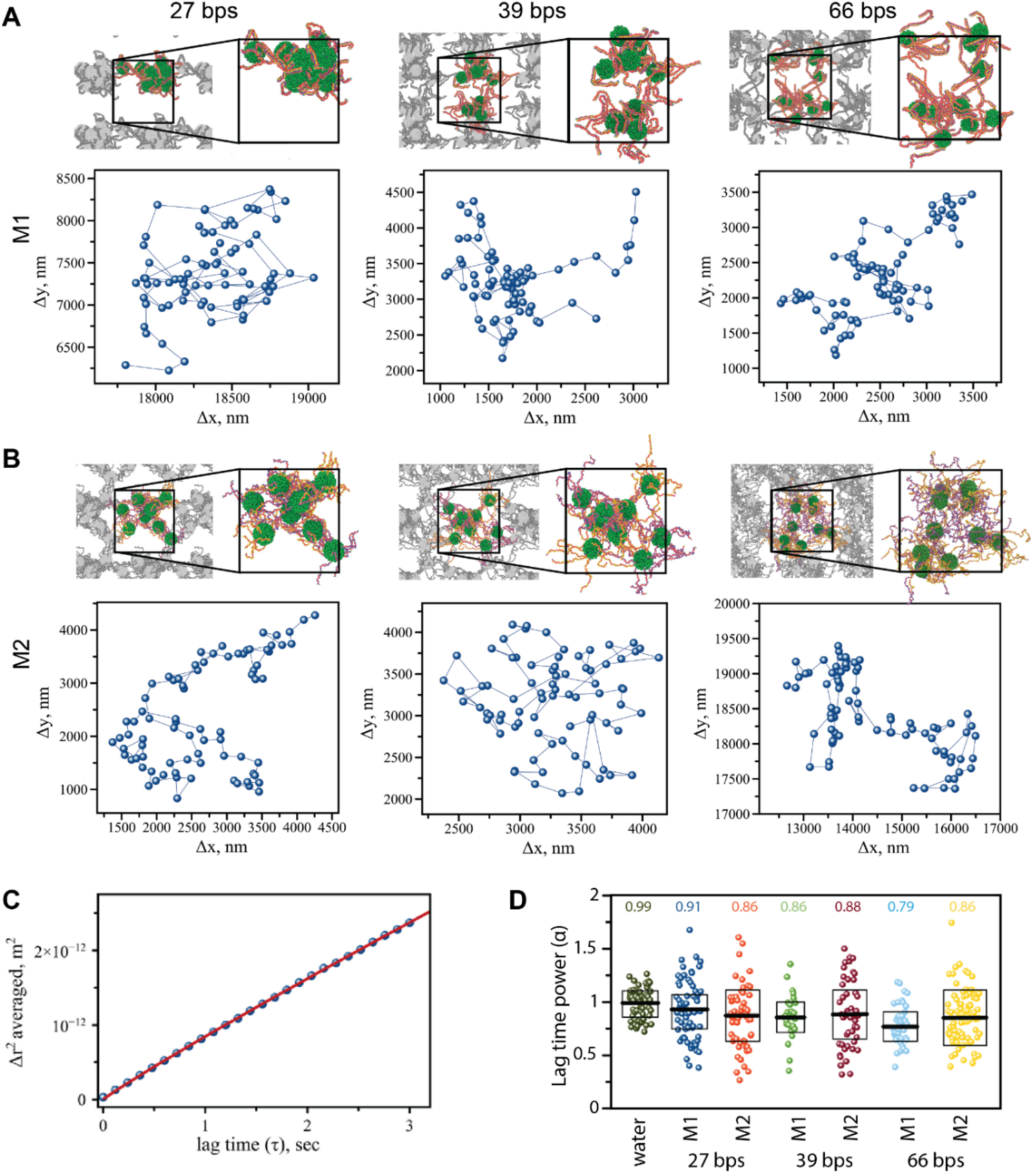
Example DPD final frames with corresponding bead tracking trajectories for (A) M1 condensates and (B) M2 condensates. (C) MSD vs lag time (τ) plot for 27 bps condensates, data points were fit (red trace) to yield the following parameters: *D* = 1.38 × 10^–13^ m^2^/s (diffusion coefficient) and α = 0.86 (power of the lag time). (D) Lag time power (α) obtained via fitting of multiple MSD plots for water and all studied condensates showing averaged α values.

We analyzed the motion of 2 μm beads (Figure S6) for the condensates with 27, 39, and 66 bps. Figure 4C shows the ensemble-averaged mean-squared displacement (MSD), < Δ*r*^2^>, plotted as a function of lag time, τ, for M2 (27 bps) condensate. The plots for all condensates were constructed as described in the experimental section using the data obtained from particle motion trajectories, shown in Figure 4A,B. The analysis was restricted to 2–3 s short lag times, while the recorded videos lasted for over 40 s. Such treatment of the data significantly increased the statistical representation of the bead’s motion on the short time scale. MSD plots were fitted, as described in Methods, yielding two parameters as fitting variables: the diffusion coefficient *D* and the lag time exponent α. The diffusion coefficient, *D*, reflects bead motion constrained by the sample’s microenvironment, while α provides insight into the viscoelastic nature of the medium. A purely viscous environment has α = 1, as indicated by a set of experiments we conducted with samples of varying glycerol concentrations to modulate sample viscosity, ranging from 0.93 × 10^–3^ Pa·s (for water) to 13.3 × 10^–3^ Pa·s (for the highest glycerol content) at room temperature (*T* ∼ 296 K). The results, presented in supporting Table S4, demonstrated the effect of viscosity change on the bead’s diffusion coefficients ranging from 2.28 × 10^–13^ m^2^/s (pure water) to 1.70 × 10^–14^ m^2^/s (highest content of glycerol). These values closely matched the expected diffusion coefficients calculated using the generalized Stokes–Einstein relation at room temperature, confirming expected behavior in viscous media.^56^

Pure water and water/glycerol mixtures consistently exhibited an α value of 1, regardless of the degree of diffusion restriction or D magnitude. In contrast, α values below 1 reflect the influence of the sample’s microstructure environment on the motion of the microbeads, likely arising from the increasing elastic characteristics of the overall viscoelastic biomolecular matrix. Figure 4D shows the values of lag power, α, for the studied condensates obtained via fitting of the MSD plots. For all three condensates, regardless of preparation method, the observed α values were below 1, suggesting a substantial elastic contribution from the condensate matrix. The diffusion coefficient was also lower than for beads diffusing in water, where D for water is 2.42 × 10^–13^ m^2^/s (calculated) and 2.28 × 10^–13^ m^2^/s (experimental), see Table S4. All condensates exhibited D < 2 × 10^–13^ m^2^/s, indicating that the condensate’s microstructure also affected the free diffusion of the beads.

**Figure 5:**
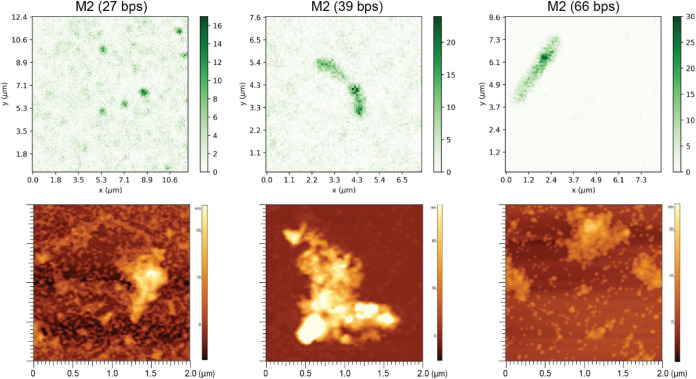
Representative fluorescence microscopy and AFM topography images of M2 condensates with 27, 39, and 66 bps DNAs.

AFM topography imaging was used to visualize condensates formed via M2 with different DNA lengths (Figure 5). The results revealed a diverse range of condensate sizes, including free QD-ssDNAs that did not participate in condensate formation. The morphology of the assembled condensates varied with size: smaller condensates predominately formed spherical structures, while larger ones often displayed elongated shapes. Overall, as was expected, the condensates exhibited nonuniform morphology with some reaching sizes up to several micrometers.

**Figure 6:**
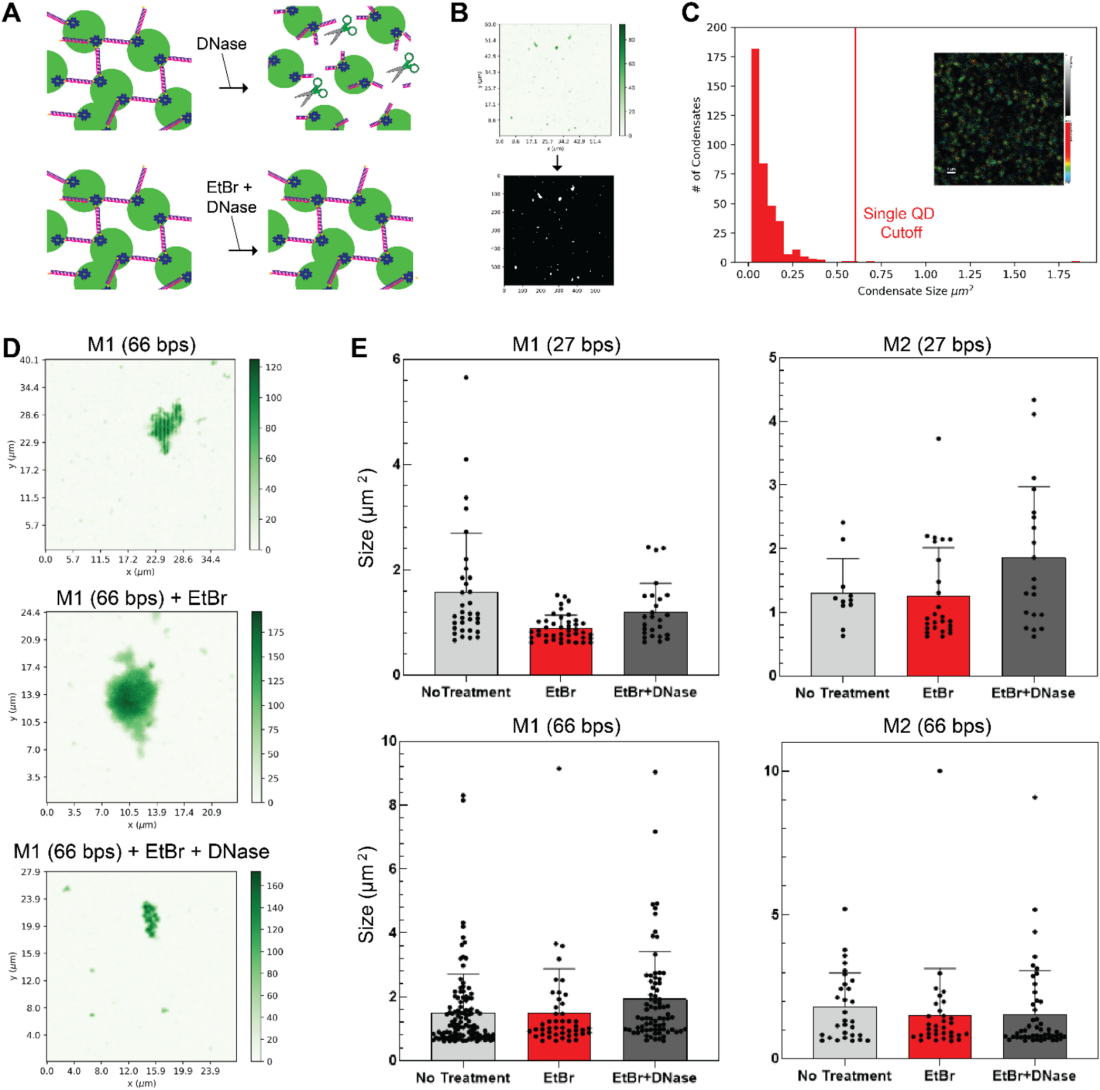
Evaluating the effect of stimuli on condensates. (A) Schematics of the two stimulus addition methods, either DNase alone, or the addition of EtBr prior to the addition of DNase, with anticipated results. (B) Example condensate image converted to a binary image by setting a threshold at four standard deviations above the average number of counts per pixel on an individual image basis with a custom Python code. (C) A representative size distribution of condensates after the addition of DNase alone. Inset is a FLIM image of a condensate sample after digestion. The scale bar is 1 μm. Single quantum dots are observed as diffraction limited spots. From control experiments imaging quantum dots alone, the cutoff size for individual QDs was found to be 0.6 μm^2^. Features below that size were omitted from the condensate size analysis. Figure S7 shows example images and FCS curves of the sample before and after digestion. (D) Representative fluorescence intensity images for condensates with no stimulus, with the addition of EtBr, and the addition of EtBr prior to DNase for M1 (66 bps). (E) The boxplots indicate the statistics for condensate sizes determined from analyzing microscopy images. Outlier condensates with a size above 10 μm^2^ (a total of 8 across all 12 experiments) were omitted from the analysis. Table S5 shows the complete statistics.

Once formed, condensates were not uniform. The number of QDs forming each of the condensates was not consistent, yielding a wide distribution of condensate sizes. The distribution is skewed toward smaller assemblies ∼1.5 μm^2^, while larger assemblies can reach an order of magnitude larger >10 μm^2^ (Figure 3D). The morphology of condensates under no extreme conditions was dependent on the linking material. We tested the ability of EtBr as an intercalating agent to protect the condensates from degradation by DNase. Figure 6A shows a schematic of the experiment. We wrote a custom Python script to create binary images (Figure 6B) from fluorescence intensity images. The threshold was set at four standard deviations above the average photon count per pixel for each image analyzed. The white spots in the binary image (Figure 6B) indicate the location of condensates. The condensate size was determined by adding the number of pixels in each white spot and converting to area using the pixel size. The physical size of one pixel, which depends on image acquisition parameters, was 0.01 μm^2^. As a control, condensates were imaged before and after digestion with DNase alone, yielding comparable FLIM and FCS (Figure S7) results when compared to QD only samples. Control images of individual quantum dots after condensates were treated with DNase revealed a lower size limit of 0.6 μm^2^ (Figure 6C). Spots smaller than that in binary images were excluded from condensate analysis. Using the thresholding methods described, we analyzed many fluorescence intensity images and extracted the condensate sizes. Representative fluorescence intensity images for M1 (66 bps) without stimulus, with EtBr, and with EtBr then DNase are shown in Figure 6D. Boxplots for M1 and M2 with 27 bps and 66 bps are shown in Figure 6E. For M1 (66 bps) and M2 (66 bps) condensates, EtBr appears to protect the condensates from digestion by DNase as the median and size distribution is similar across all conditions. For M2 (27 bps), the addition of EtBr and DNase slightly increases the median and size distribution, suggesting a swelling effect due to the intercalating stimulus.

The addition of DNase into any of the condensates resets the lag time power, α, to larger values bringing them closer to 1, as summarized in Table 2. This observation suggests that samples return to a more viscous state upon removal of the cross-linked microstructure within the condensate sample. Although the diffusion coefficients also increase across all three samples, they do not reach the value of D corresponding to pure water. These observations strongly suggest that while DNase effectively removes the microstructure, the samples remain largely viscous, most likely attributed to all the components of the condensates still present in the sample. This demonstrates that external triggers such as DNase can modify the characteristics of the condensate and significantly influence their local mechanical properties.

**Table 2:** Fitting Parameters of the MSD Plots for Water, M2 Condensates, and M2 Condensates with Addition of DNase

sample M# (# of bps)	lag time power, α	diffusion coefficient, *D* (m^2^/s)
water	0.99	2.28 × 10^–13^
M2 (27)	0.86	1.38 × 10^–13^
M2 (39)	0.88	1.54 × 10^–13^
M2 (66)	0.86	1.40 × 10^–13^
M2 (27) + DNase	0.99	1.57 × 10^–13^
M2 (39) + DNase	0.98	1.86 × 10^–13^
M2 (66) + DNase	0.97	1.84 × 10^–13^

**Figure 7:**
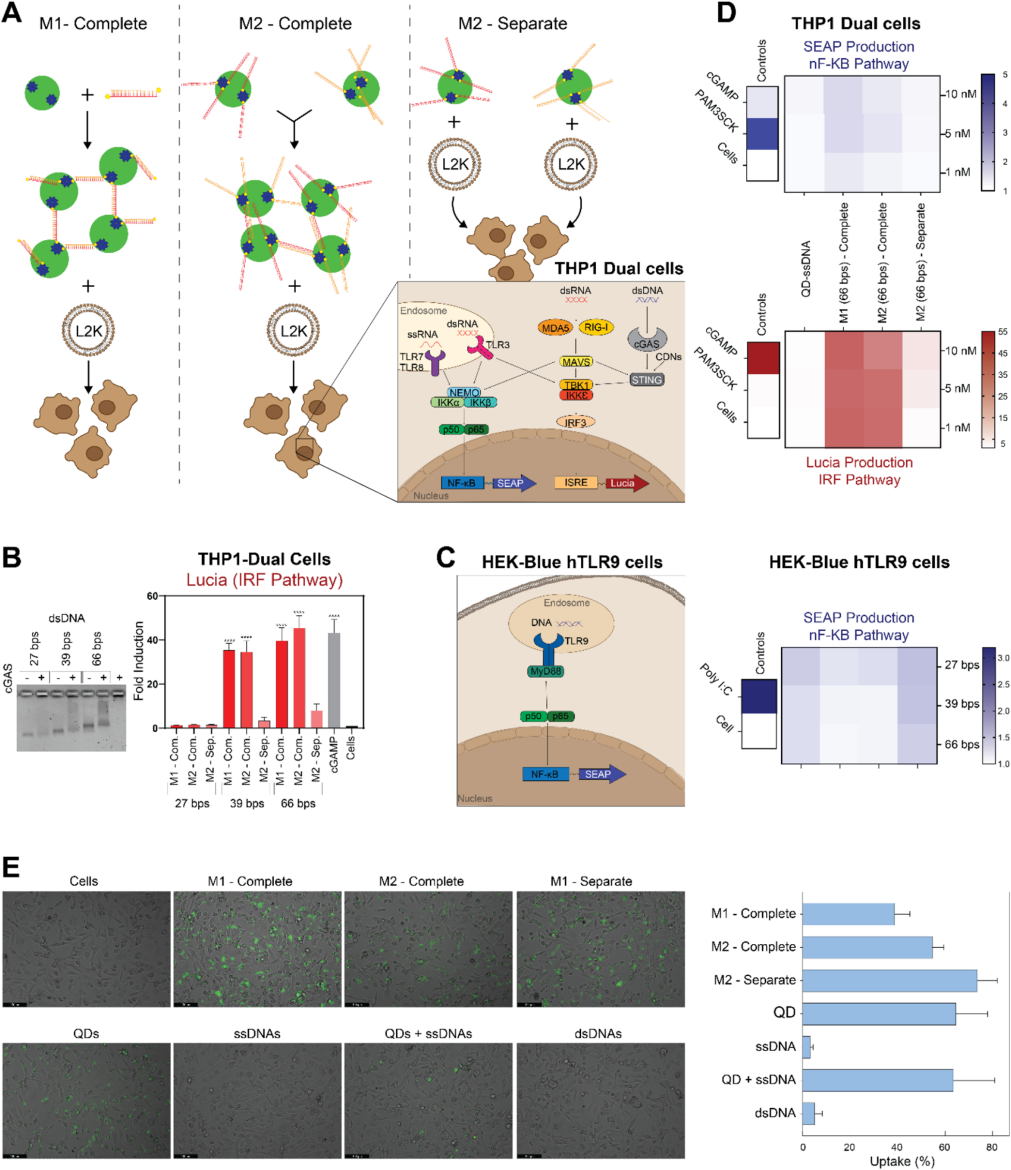
Evaluation of the immunostimulatory properties. (A) depicts the methodology of synthesis of condensates evaluated on (B and D) THP1-Dual Cells and (C) Human TLR9 Cells with condensates composed of dsDNA of lengths 27, 39, and 66 bps. (B) To further evaluate the mechanism of activation of IRF pathway, binding of dsDNA at different lengths were evaluated against cGAS protein. (D) Because the cGAS protein was bound to the 66 bps dsDNAs, the condensates formed with 66 bps dsDNAs were used to evaluate SEAP and Lucia production at various concentrations of complexes formed. ssDNA of 66 bp length was used as a control to show that the QD-ssDNA remained undetected at the same length. (E) Cellular uptake of the condensates with dsDNA of length 66 bps imaged using fluorescence microscope at a 20× magnification (more 10× microscopy images are shown in Figure S8) and quantified using flow cytometry and presented as mean ± SD, *n* = 2.

Several experiments with human cell cultures were conducted to evaluate the ability of preformed condensates to be transfected into mammalian cells or formed intracellularly, as well as to assess their immunostimulatory potential. Cell viability was assessed in all experiments to eliminate the variability of immunorecognition for different experimental methods. The results showed no significant cell death among the different condensates (Figure S8). When used with reporter cell lines (Figures 7 and S8), neither cell line evaluating SEAP activity following the NF-κB pathway activation showed significant immunostimulation. Conversely, Lucia excretion following the activation of the Interferon Regulatory Factor (IRF) pathway, specifically IRF3, showed significant immunostimulation from transfected condensates produced both via M1 and M2. Furthermore, QD-ssDNA samples of the same length did not show activation, indicating that when cotransfected, the QD-ssDNA remains undetected (Figure 7D). Based on the results and due to the origin of the material, it was hypothesized that the activation of the IRF3 pathway occurred due to dsDNA interaction with cGAS in a length-dependent manner,^57,58^ which lead to the next section where dsDNA binding to cGAS was evaluated. While purified cGAS was able to form a complex with all dsDNA, as indicated by the reduced band intensity (Figure 7B), it was noticeable that the mobility of 39 bps and 66 bps dsDNAs was reduced more than that of 27 bps dsDNA. Therefore, it was concluded that the condensates formed from longer dsDNAs could activate the cGAS-STING signaling pathway. Furthermore, as expected, a delivery carrier was required for condensate uptake within the cells, as the cargo could not be internalized without a carrier (Figure S9). When complexed with carriers, the uptake percentage was significantly higher (Figure 7E), as demonstrated by fluorescence microscopy images alongside flow cytometry analysis, providing both qualitative and quantitative validation. These discoveries laid a foundation for guiding the design principles to assemble the biomolecular condensates to either facilitate the therapeutic immunomodulation of cGAS-STING pathway or promote immune-quiescent delivery for potential uses across a spectrum of biomedical applications.

## Conclusions

In conclusion, biomolecular condensates are crucial for cellular functions, allowing vital biochemical reactions to occur with controlled reaction rates. This study utilized a variety of computational and experimental approaches to study the design and characterization of synthetic condensates and predict their structure–activity relationship. The assembly method and DNA length were identified as key factors governing the condensates’ morphology, size, and viscoelastic properties. DPD simulations revealed that M1 produced larger, compact globular clusters, while M2 formed smaller, more diffuse structures. All computational findings were corroborated by confocal microscopy, which showed that M1 consistently produced larger, globular condensates across all DNA lengths, while M2 formed smaller, less compact clusters with irregular morphologies. Stimuli responsiveness studies provided further insights into the structural adaptability of these condensates, and microrheology experiments demonstrated that DNase digestion disrupted the cross-linked microstructures of the condensates, shifting their mechanical properties toward a more viscous state with lower elastic contribution to overall viscoelastic behavior. EtBr intercalation induced mild swelling in condensates, shifting their morphology from linear to globular while also protecting the structures from complete digestion by nucleases.

Furthermore, structural differences in the biomolecular condensates dictated by the length of DNA components allowed for regulated activation of the IRF pathway, and mechanistic studies revealed the involvement of cGAS in condensates’ immune recognition. Collectively, this data advanced our understanding of the immunological properties of hybrid nanomaterials and laid a foundation to guide the formation of biomolecular condensates with desired immunostimulation profiles following their intracellular delivery.

The future applications of this technology are vast. Synthetic condensates can be engineered for intra- and extracellular use, designed to encapsulate specific cargo that can be released in response to stimuli such as nucleases, changes in pH, or variations in salt concentration. This selective cargo release and controlled immunorecognitions offer the potential to minimize off-target effects and improve therapeutic precision. The demonstrated ability to predict and control condensate size, shape, and mechanical properties paves the way for the development of tailored biomaterials for applications in synthetic biology, targeted drug delivery, and immunostimulation. This study establishes a foundational framework for the programmable design of biomolecular condensates with customizable properties and dynamic responsiveness.

## Supporting Information Available

Simulations for M1(39 bp) condensates formation (MP4)
A list of all nucleic acid sequences used in this work, DPD methods, supporting tables, and supporting figures detailing simulation results, fluorescence microscopy and decay, motion trajectories, and additional cell culture experiments (PDF)
Simulations for M2(39 bp) condensates formation (MP4)
