# Supplementary material for: Design and Characterization of DNA-Driven Condensates: Regulating Topology, Mechanical Properties, and Immunorecognition
Source: ACS Appl Mater Interfaces. 2025 Apr 1;17(15):22322–36. doi: 10.1021/acsami.5c00428 (PMC12012714; doi:10.1021/acsami.5c00428)
Supplement: Supplementary file 2 — am5c00428_si_002.pdf [file am5c00428_si_002.pdf]

Supporting information for

Design and Characterization of DNA-Driven Condensates: Regulating Topology, Mechanical Properties, and Immunorecognition

Elizabeth Skelly[1+], Christina J. Bayard[2+], Joel Jarusek[3+], Benjamin Clark[4+], Laura P. Rebolledo[1], Yasmine Radwan[1], Phong Nguyen[1], Melanie Andrade-Muñoz[1], Thomas A. Deaton[2], Alexander Lushnikov[3], Sharonda J. LeBlanc[4*], Alexey V. Krasnoslobodtsev[3*], Yaroslava G. Yingling[2*], Kirill A. Afonin[1*]

[1]Chemistry and Nanoscale Science Program, Department of Chemistry, University of North Carolina at Charlotte, Charlotte, NC, 28223, USA

[2]Department of Materials Science and Engineering, North Carolina State University, Raleigh, NC, 27695, USA

[3]Department of Physics, University of Nebraska Omaha, Omaha, NE, 68182, USA

[4]Department of Physics, North Carolina State University, Raleigh, NC, 27695-8202, USA

[+]E.S., C.J.B., J.J. and B.C. contributed equally to this paper.

*Correspondence should be addressed to: kafonin@charlotte.edu; ygyingli@ncsu.edu; akrasnos@unomaha.edu; sleblan@ncsu.edu.

Sequences used in this project

Name	Sequence
27.1	/5Biosg/CCCAAGGTCCGAACGACATTAATGGGT
27.2	/5Biosg/ACCCATTAATGTCGTTCGGACCTTGGG
39.1	/5Biosg/GGAGACCGTGACCGGTGGTGCAGATGAACTTCAGGGTCA
39.2	/5Biosg/TGACCCTGAAGTTCATCTGCACCACCGGTCACGGTCTCC
66.1	/5Biosg/GGAGACCGTGACCGGTGGTGCAGATGAACTTCAGGGTCACGGTGGTGCAGATGAACTTCAGGGTCA
66.2	/5Biosg/TGACCCTGAAGTTCATCTGCACCACCGTGACCCTGAAGTTCATCTGCACCACCGGTCACGGTCTCC

Further DPD Methods

The DPD pairwise potential energy [*Equation S1*] consists of a conservative force, F^C_{ij}; dissipative force, F^D_{ij}; random force, F^R_{ij}, and a harmonic bonded spring force (F^S_{ij}),

$$\frac{dr_i}{dt} = v_i, m_i \frac{dv_i}{dt} = \sum_{j \neq i} f_{ij}\, , f_{ij} = \left(F^C_{ij} + F^D_{ij} + F^R_{ij}\right) + F^S_{ij}$$

Equation S1

where r_i, v_i, and m_i are the position, velocity, and mass of bead *i*. The conservative, dissipative, and random forces are:

$$F^C_{ij} = \begin{cases} a_{ij}\left(1 - \frac{r_{ij}}{r_c}\right)\bar{r}_{ij}, & r_{ij} < r_c \\ 0, & r_{ij} > 0 \end{cases}$$

Equation S2

$$F^D_{ij} = -\gamma\omega^D(r_{ij})(\bar{r}_{ij} \cdot v_{ij})\bar{r}_{ij}$$

Equation S3

$$F^R_{ij} = \sigma_D \omega^R(r_{ij})\theta_{ij}\Delta t^{-\frac{1}{2}}\bar{r}_{ij}$$

Equation S4

where a_{ij} is the maximum repulsion; r_{ij} is the distance between *i* and *j*; $v_{ij} = v_i - v_j$; and lastly $\bar{r}_{ij}$ is the unit vector along *j* to *i*. Δt is the timestep, γ and σ_D are the dissipative and random force coefficients respectively.

The dissipative force coefficient, γ, can be found through

$$\gamma = \frac{\sigma_D^2}{2k_B T}$$

Equation S5

Note that $\theta_{ij}(t)$ is a symmetric random number; and w^D and w^R are related to each other by:

$$w^D(r_{ij}) = \left[w^R(r_{ij})\right]^2 = \begin{cases} \left(1 - \frac{r_{ij}}{r_c}\right)^2, & r_{ij} < r_C \\ 0, & r_{ij} > r_C \end{cases}$$

Equation S6

The additional bonded spring force is a harmonic interaction with a spring constant:

$$F^S_{ij} = Cr_{ij}$$

Equation S7

Established by Li et al., the implicit ionic strength of the solvent in DPD can be related to the second virial coefficient:

$$v = v_A + \frac{\alpha^2}{c_s}$$

Equation S8

where v_A is the non-electrostatic contribution, α is the degree of ionization and c_s is the ionic strength of the solvent. This establishes the inverse relationship to solvent ionic strength and can be communicated in the polyelectrolyte bead self-repulsion:

$$a_{AA} = a_{ii} + a_{elec}$$

Equation S9

where a_{ii} = 25 when density is 3, as established by Groot and Warren[31] and a_{elec} is the inverse referent to the solvent ionic strength. For this study, a_{AA}varied from 40 – 90.

Supporting Tables

Table S1. Key LAMMPS simulation parameters.

Category	Parameter / Command	Setting / Value	Description
**Force Field**	**Pair Style**	dpd/gpu 1.0 1.0 34387234	Uses a DPD potential with GPU acceleration; parameters: temperature=1.0, cutoff=1.0, seed=34387234.
	**Pair Coefficients**	See Table S2	Defines interaction strengths and cutoffs between atom types.
	**Bond Style**	harmonic	Employs a harmonic potential for bond stretching.
	**Bond Coefficients**	bond_coeff for types 1, 2, 3: 100.0 0.1	Uniform force constant (100.0) and equilibrium bond length (0.1) for specified bonds.
	**Angle Style**	harmonic	Uses a harmonic potential for angle bending.
	**Angle Coefficients**	Type 1: 20.0 150.0; Type 2: 15.0 100.0	Sets parameters (stiffness coefficient, angle) for backbone–backbone (20.0, 150.0) and backbone–base (15.0, 100.0) interactions.
**Thermodynamic Ensemble**	**Fix 1 (Rigid Body Integration)**	fix 1 all rigid/nve group [qd_1–qd_10]	Applies rigid body NVE integration to individual QD models.
	**Fix 2 (NVE Integration)**	fix 2 notqd nve	Implements NVE integration (typical for DPD) for all other particles.
**dyBonding (dsDNA)**	**Fix 3 (Bond Creation)**	fix 3 qddsdna bond/create 1 8 9 0.8 3 iparam 1 6 jparam 1 7	Enables dynamic bond creation between "streptavidin" and "biotin" bead types that are within 0.8r_c of each other. Changes bead type after forming bond.
**dyBonding (ssDNA)**	**Fix 3-5 (Bond Creation)**	fix 3 qddsdna bond/create 1 8 13 0.8 3 iparam 1 6 jparam 1 7<br>fix 4 qddsdna bond/create 1 9 12 0.8 3 iparam 1 6 jparam 1 7<br>fix 5 qddsdna bond/create 1 10 11 0.8 3 iparam 1 6 jparam 1 7	Enables dynamic bond creation between corresponding beads within 0.8r_c of each other on ssDNA strands. The dyBonding schemes are shown in Figure 2.
**Equilibration**	**Initial Velocity Setup**	velocity all create 1.0 492849 dist gaussian	Assigns initial velocities (Gaussian distribution) at a target temperature of 1.0.
	**Equilibration Timestep**	0.005 (dsDNA), 0.002 (ssDNA)	Time integration step used during the equilibration phase.
	**Equilibration Run 1**	1000 steps	First equilibration segment after velocity initialization.
	**Equilibration Run 2**	200 steps	Additional equilibration following a second velocity re-initialization.
**Production**	**Production Timestep**	0.01 (dsDNA), 0.004 (ssDNA)	Increased time step for production dynamics.
	**Production Runs**	$8x10^5$ steps	
	**Restart Frequency**	Write to restart file every $5x10^4$ steps	Periodic restart files for simulation recovery.

Table S2. Non-Bonded Interactions.

**Interaction Description**	**a_{ij} (M1)**	**a_{ij} (M2)**
backbone to backbone	90	90
backbone to backbone (high salt)	40	40
backbone to nucleobases	40	40
backbone to nucleobases (high salt)	30	30
backbone to QD	15	15
backbone to water	25	25
nucleobase type A to type A	22	22
nucleobase type A to type B	5	5
nucleobase to QD	27	27
nucleobase to water	27	27
QD to water	25	25
water to water	25	25

Table S3. Statistical analysis of DBSCAN clustering performed on final frame of high salt concentration DPD simulations.

<table>
<tr><th rowspan="2">Sample Method (# of bps)</th><th rowspan="2">Number of clusters</th><th rowspan="2">Avg. distance to cluster centroid (averaged across clusters)</th><th colspan="3">Welch two sample t-test</th><th>Wilcoxon rank sum test</th></tr>
<tr><th>Test statistic (t)</th><th>95% confidence interval</th><th>P-value</th><th>P-value</th></tr>
<tr><td>M1 (27)</td><td>5</td><td>5.69</td><td rowspan="2">-71.73</td><td rowspan="2">(-3.95, -3.74)</td><td rowspan="2">2.2 x 10^{-16}</td><td rowspan="2">2.2 x 10^{-16}</td></tr>
<tr><td>M2 (27)</td><td>1</td><td>9.53</td></tr>
<tr><td>M1 (39)</td><td>2</td><td>16.63</td><td rowspan="2">85.89</td><td rowspan="2">(4.90, 5.13)</td><td rowspan="2">2.2 x 10^{-16}</td><td rowspan="2">2.2 x 10^{-16}</td></tr>
<tr><td>M2 (39)</td><td>1</td><td>11.61</td></tr>
<tr><td>M1 (66)</td><td>4</td><td>11.03</td><td rowspan="2">-84.55</td><td rowspan="2">(-4.70, -4.49)</td><td rowspan="2">2.2 x 10^{-16}</td><td rowspan="2">2.2 x 10^{-16}</td></tr>
<tr><td>M2 (66)</td><td>1</td><td>15.62</td></tr>
</table>

Table S4. Theoretical and experimental values of diffusion coefficients for glycerol/water viscous mixtures. Theoretical values of diffusion coefficients were calculated using the Stokes-Einstein relationship for a spherical particle: $D = \frac{k_B T}{6\pi\eta a}$.

**Glycerol/water mixtures (w%), Viscosity, Pa·s**	**Diffusion Coefficient, D (m^2/s)**	
	Theoretical	Experimental
0%/100% (0.933 $\times10^{-3}$)	2.42×10^{-13}	2.28×10^{-13}
18%/82% (1.66×10^{-3})	1.36×10^{-13}	1.50×10^{-13}
32.2%/67.8% (2.5×10^{-3})	9.04×10^{-14}	8.25×10^{-14}
44.2%/55.8% (4.65×10^{-3})	4.86×10^{-14}	5.22×10^{-14}
64.5%/35.5% (13.2×10^{-3})	1.71×10^{-14}	1.70×10^{-14}

Table S5. Statistics used to generate box plots in Figure 6 in the main text.

Method (# of bps)	Treatment	Mean (μm^2)	Std. Dev. (μm^2)	Median (μm^2)	Number of condensates
M1 (66)	None	1.49	1.22	1.1	116
	EtBr	1.5	1.38	1.03	46
	EtBr/DNase	1.92	1.49	1.35	76
M2 (66)	None	1.8	1.19	1.307	30
	EtBr	1.5	1.64	1.03	33
	EtBr/DNase	1.546	1.518	0.865	50
M1 (27)	None	1.587	1.105	1.167	33
	EtBr	0.895	0.245	0.830	42
	EtBr/DNase	1.199	0.542	1.076	26
M2 (27)	None	1.306	0.534	1.190	11
	EtBr	1.26	0.76	0.88	26
	EtBr/DNase	1.854	1.119	1.453	20

Supporting Figures

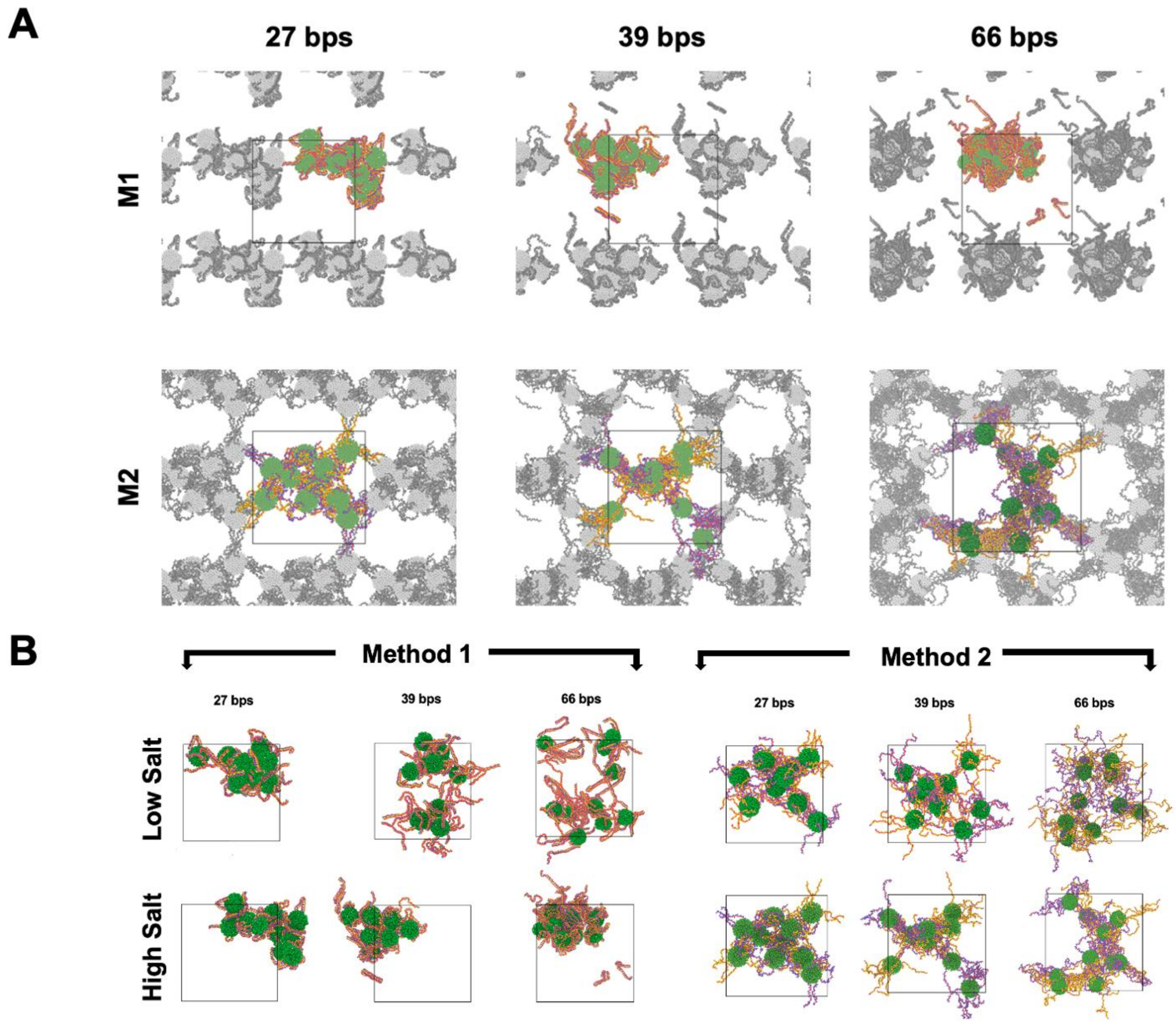

Figure S1. (**A**) Final frames from 8 x 10^5 steps DPD simulations under high salt conditions. (**B**) Comparison of final frame for low and high salt concentration for both methods.

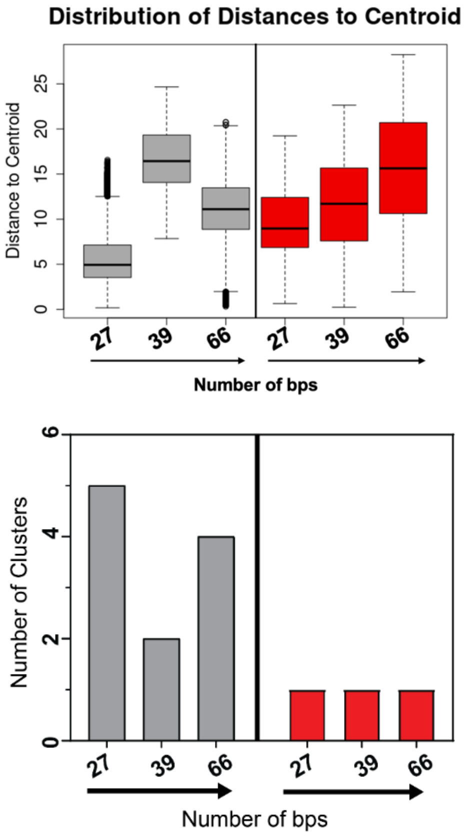

Figure S2. Distribution of distances to centroid (*above*) for M1 (grey) and M2 (red) and final number of clusters for each DPD simulation (*below*) under high salt conditions.

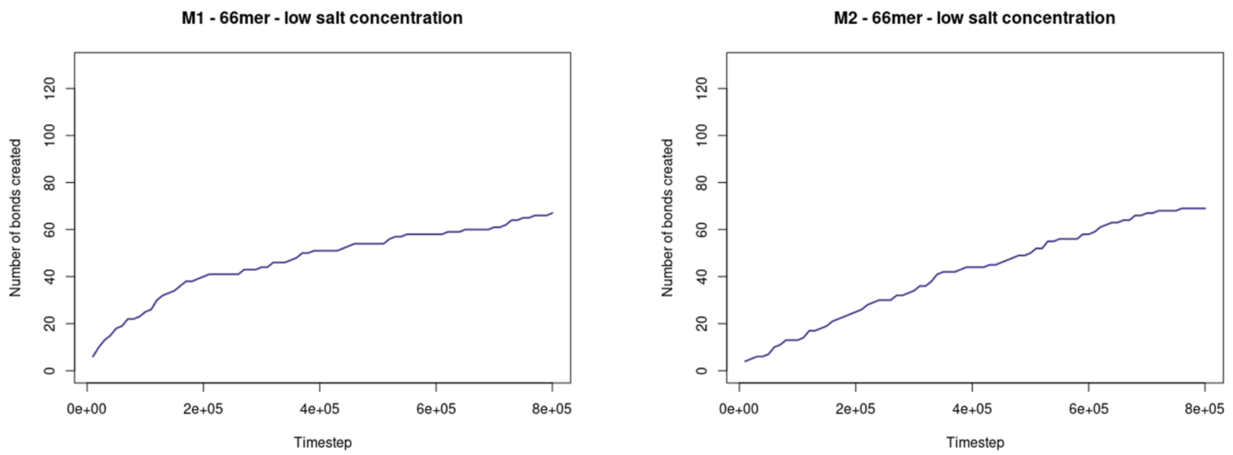

Figure S3. Number of bonds created through dyBonding for Methods 1 (*left*) and 2 (*right*) throughout the simulations. Convergence of simulations was dictated by a plateau in the number of bonds created for each simulation.

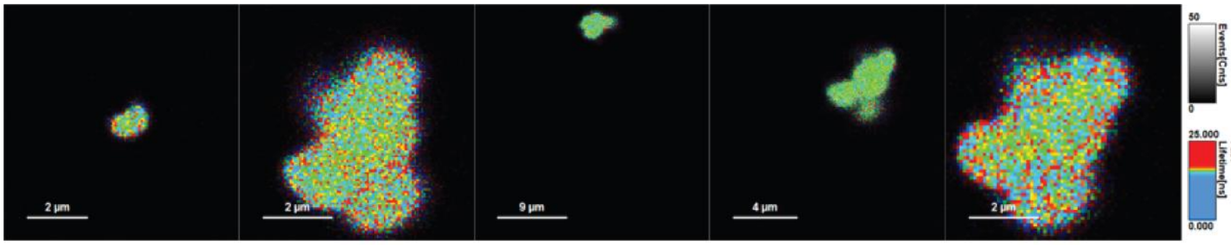

$\tau_1 = 25.0 \pm 4.2\, ns$ $\tau_2 = 2.3 \pm 0.5\, ns$

$\tau_1 = 16.1 \pm 0.1\, ns$ $\tau_2 = 1.4 \pm 0.1\, ns$

$\tau_1 = 16.0 \pm 0.3\, ns$ $\tau_2 = 1.4 \pm 0.1\, ns$

$\tau_1 = 16.0 \pm 0.1\, ns$ $\tau_2 = 1.24 \pm 0.06\, ns$

$\tau_1 = 15.1 \pm 0.1\, ns$ $\tau_2 = 0.4 \pm 0.1\, ns$

Figure S4. FLIM images of M1 (66 bps) in phosphate buffered saline (PBS, NaCl: 137 mM, KCl: 2.7 mM, Na2HPO4: 10 mM, KH2PO4: 1.8 mM, pH 7.4). The two fluorescence lifetime components from a bi-exponential fit are indicated below each image with corresponding fit error. Other FLIM measurements were conducted in 20 mM Tris HCl, 5 mM $MgCl_2$, 100 mM sodium acetate, pH 7.5. The fluorescence lifetimes of the condensates in PBS appear to be longer compared to representative images under those conditions.

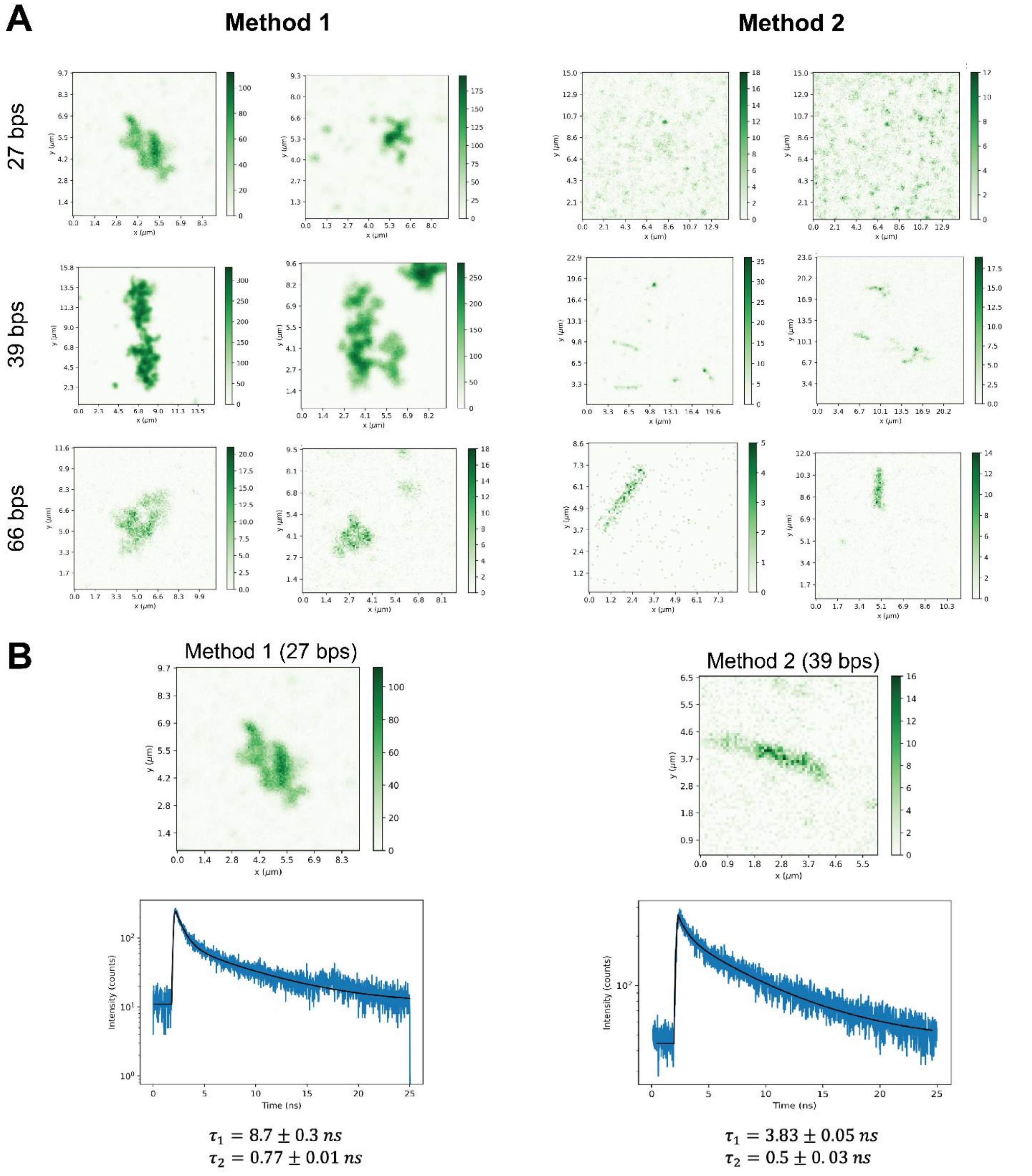

Figure S5. (**A**) Representative fluorescence intensity images for 2 condensate areas per sample type. (**B**) Two representative fluorescence intensity images and the corresponding excited state decays generated from binning the photon arrival nanotimes of all photons from the image. The experimental decay is shown in blue and the fit to a bi-exponential function is the black curve. The two lifetime components extracted from the fits are indicated below each plot.

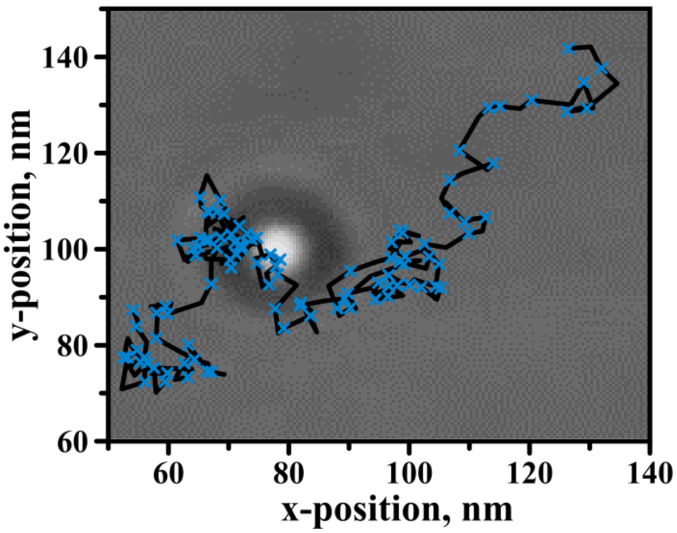

Figure S6. Image of a bead and its corresponding tracked motion trajectory.

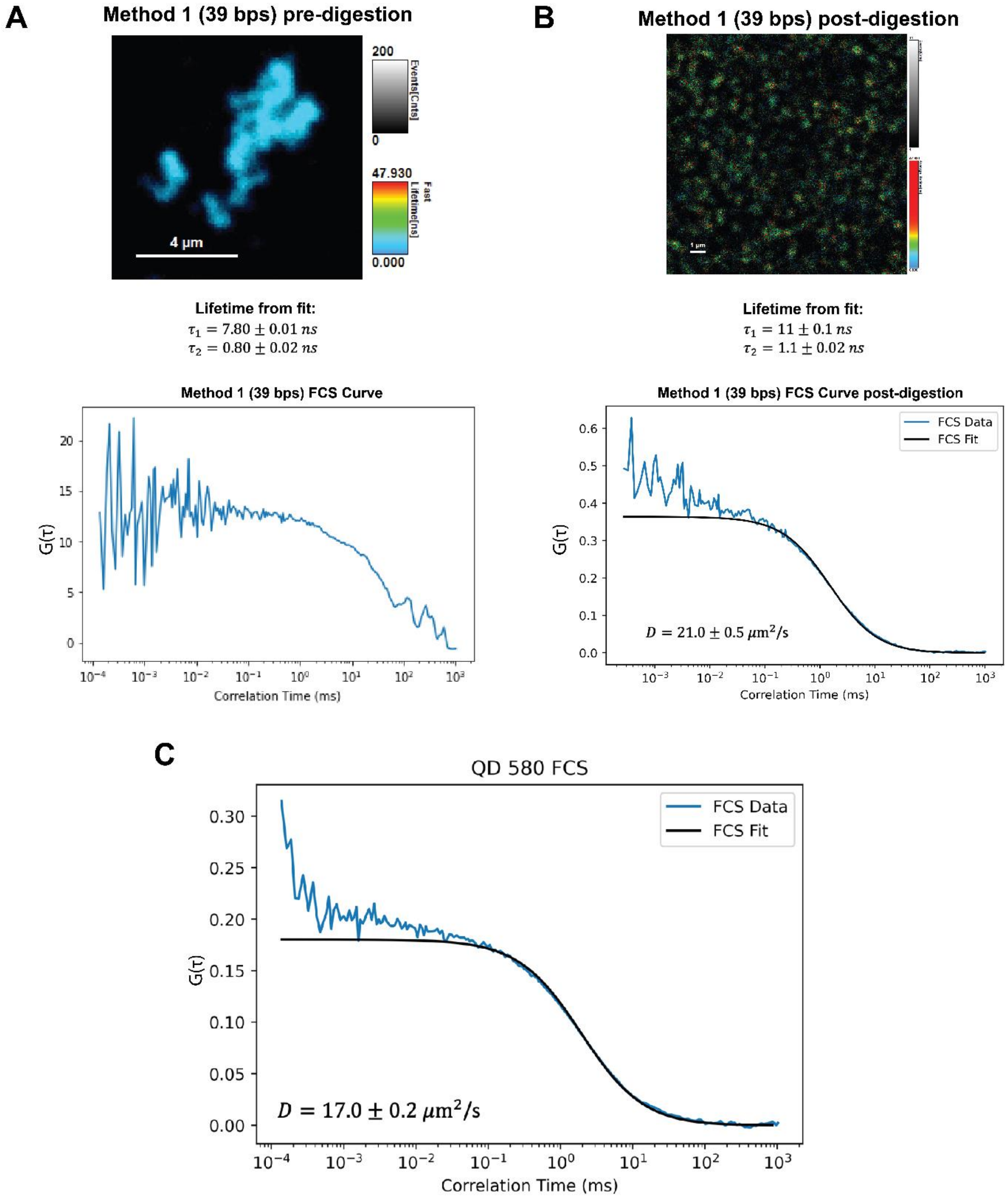

Figure S7. (**A**) Representative FLIM image of M1 (39 bps) condensate before digestion with DNase. The color of each pixel indicates the fluorescence lifetime at that pixel (average photon arrival nanotime). An excited state decay was generated and fit to a bi-exponential function (not shown). The two extracted lifetime components with fit errors are shown below the image. The FCS signal was also measured for a 10 nM solution of condensates. Prior to digestion, the size of the condensates is much larger than the confocal volume and the characteristic decay at the

millisecond time for diffusing particles is absent. (**B**) Representative FLIM image of M1 (39 bps) condensate after digestion with DNase. The color of each pixel indicates the fluorescence lifetime at that pixel (average photon arrival nanotime). An excited state decay was generated and fit to a bi-exponential function (not shown). The two extracted lifetime components with fit errors are shown below the image. The FCS signal was also measured for a 10 nM solution of condensates after digestion. After digestion, the size of the condensates is small compared to the confocal volume and the decay could be fit to a pure diffusion model (G(τ), Equation S10 below) to extract the diffusion coefficient shown on the plot. (**C**) The FCS curve for a control sample of QD 580 with the extracted diffusion coefficient from a pure diffusion model fit to Equation S10.

$$G(\tau)_{pure\ diffusion} = \frac{1}{N} \cdot \frac{1}{1+\frac{\tau}{\tau_D}} \cdot \sqrt{\frac{1}{1+\frac{\tau}{\tau_D} \cdot \frac{{\omega_0}^2}{{z_0}^2}}}$$

Equation S10

, where N is the number of molecules in the confocal volume at any instant, τ_D is the characteristic diffusion time of the species, ω_0 is the diameter of the laser beam waist, and z_0 is the axial length of the laser beam. The ratio ${}^{z_0}/_{\omega_0}$ is the confocal volume aspect ratio, represented by κ. The aspect ratio was estimated by measuring several dyes with known diffusion coefficients, *D*, and imaging 100 nm beads. The diffusion coefficient was then calculated as $D = \omega_0^2/4\tau_D$.

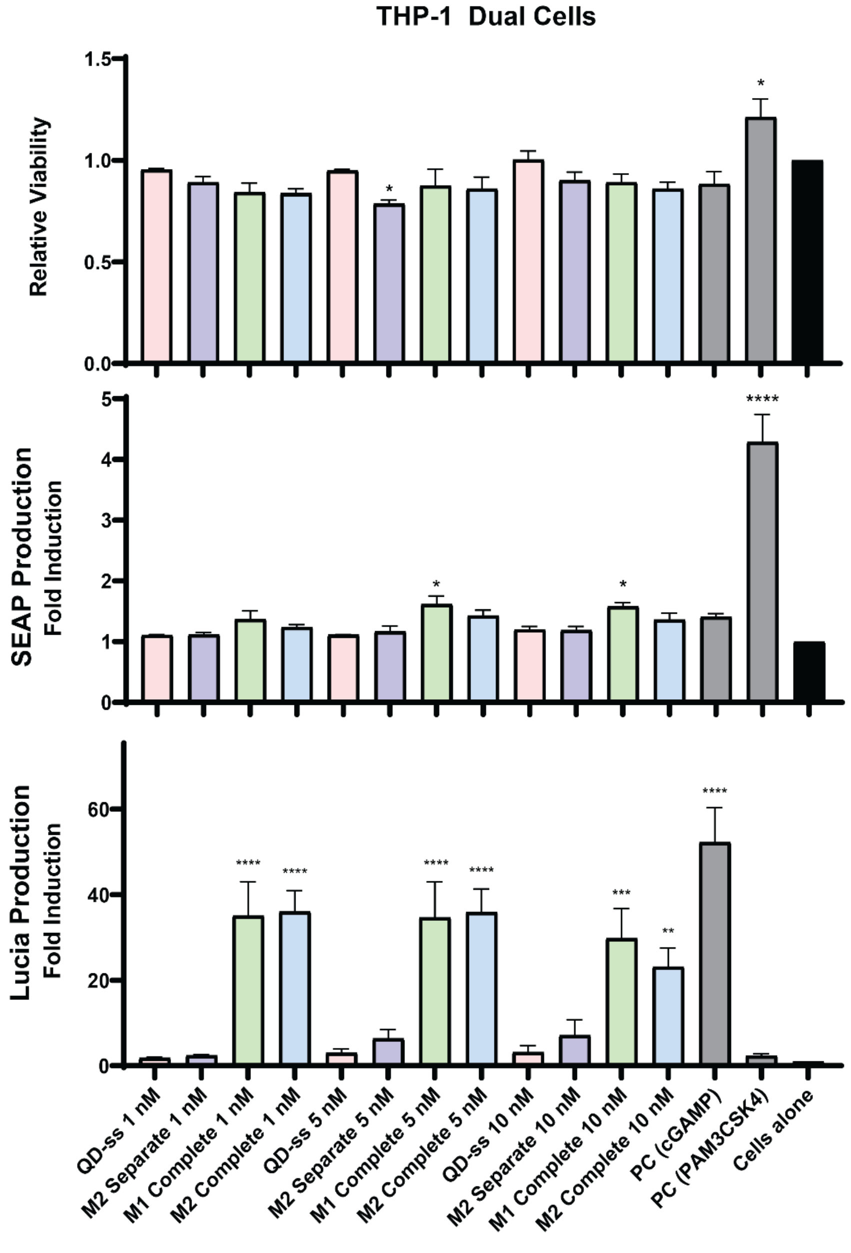

Figure S8. Relative viability and SEAP/Lucia production fold induction. Indication of N digits after the decimal: *, **, ***, or ****. No * indicates not significant.

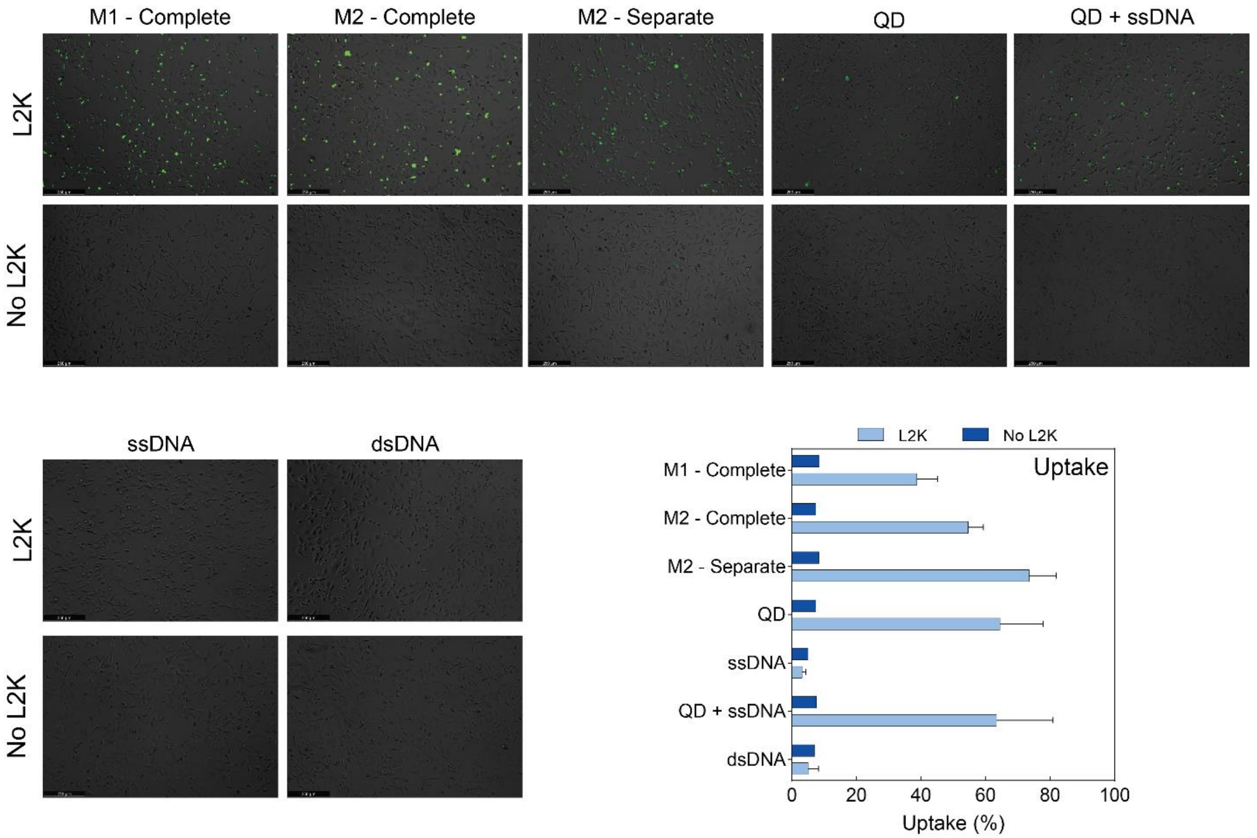

Figure S9. Condensates cellular uptake in MDA-MB-231 cells. Merged GFP and brightfield images captured using a Leica fluorescence microscope at 10X magnification show the uptake of 66 bps condensates in MDA-MB-231 cells, with and without Lipofectamine 2000 (L2K). Graph shows OVERTON flow cytometry analysis quantifying the percentage of condensate uptake in MDA-MB-231 cells treated with 66 bps condensates, either complexed with L2K or unassisted. Uptake efficiency is significantly enhanced in the presence of L2K. Data are presented as mean ± SD, with L2K treatments (n=2) and no L2K treatments (n=1).
